# Molecular-Based Fluorescent Nanoparticles Built from Dedicated Dipolar Thienothiophene Dyes as Ultra-Bright Green to NIR Nanoemitters

**DOI:** 10.3390/molecules21091227

**Published:** 2016-09-14

**Authors:** Cristiano Mastrodonato, Paolo Pagano, Jonathan Daniel, Michel Vaultier, Mireille Blanchard-Desce

**Affiliations:** Institut des Sciences Moléculaires (UMR 5255 CNRS), Université de Bordeaux, 33400 Talence, France; cristiano-matteo.mastrodonato@u-bordeaux.fr (C.M.); paolo.pagano@u-bordeaux.fr (P.P.); jonathan.daniel@u-bordeaux.fr (J.D.); michel.vaultier@u-bordeaux.fr (M.V.)

**Keywords:** fluorescence, thienothiophene, nanoparticles, dipolar chromophores, two-photon absorption

## Abstract

Fluorescent Organic Nanoparticles (FONs), prepared by self-aggregation of dedicated dyes in water, represent a promising green alternative to the toxic quantum dots (QDs) for bioimaging purposes. In the present paper, we describe the synthesis and photophysical properties of new dipolar push-pull derivatives built from thieno[3,2-*b*]thiophene as a π-conjugated bridge that connects a triphenylamine moiety bearing various bulky substituents as electron-releasing moiety to acceptor end-groups of increasing strength (i.e., aldehyde, dicyanovinyl and diethylthiobarbiturate). All dyes display fluorescence properties in chloroform, which shifts from the green to the NIR range depending on the molecular polarization (i.e., strength of the end-groups) as well as a large two-photon absorption (TPA) band response in the biological spectral window (700–1000 nm). The TPA bands show a bathochromic shift and hyperchromic effect with increasing polarization of the dyes with maximum TPA cross-section reaching 2000 GM for small size chromophore. All dyes are found to form stable and deeply colored nanoparticles (20–45 nm in diameter) upon nanoprecipitation in water. Although their fluorescence is strongly reduced upon aggregation, all nanoparticles show large one-photon (up to 10^8^ M^−1^·cm^−1^ in the visible region) and two-photon (up to 10^6^ GM in the NIR) brightness. Interestingly, both linear and non-linear optical properties are significantly affected by interchromophoric interactions, which are promoted by the molecular confinement and modulated by both the dipolar strength and the presence of the bulky groups. Finally, we exploited the photophysical properties of the FONs to design optimized core-shell nanoparticles built from a pair of complementary dipolar dyes that promotes an efficient core-to-shell FRET process. The resulting molecular-based core-shell nanoparticles combine large two-photon absorption and enhanced emission both located in the NIR spectral region, thanks to a major amplification (by a factor of 20) of the core fluorescence quantum yield. These novel nanoparticles, which combine huge one-and two-photon brightness, hold major promise for in vivo optical bioimaging.

## 1. Introduction

In recent years, luminescent nanoparticles have emerged as an active research field with applications in various fields including optoelectronics and imaging. So far, the most popular luminescent nanoparticles are essentially inorganic either based on metals, semiconductors or metal oxides [1,2]. Among those, semiconductor-based Quantum Dots (QDs) have attracted a lot of interest due to their unique electronic and optical properties (including high brightness, photostability, tunability). These inorganic nanoparticles are widely used in solar cells [3,4,5] and for bioimaging purposes [6,7]. Yet, they suffer from several drawbacks such as toxicity and blinking and raise a number of questions with respect to environmental issues (clearance, biodegradability). In particular, the presence of heavy metals as components of the brightest QD is still a limitation, in particular for biomedical applications [8,9]. In this perspective, luminescent organic nanoparticles offer a promising alternative. Among them, *molecular-based* Fluorescent Organic Nanoparticles (so-called FONs) have attracted a lot of interest in view of their versatility [10,11]. Due to their molecular nature, these nanoparticles offer a paramount bottom-up approach.

In particular, molecular engineering of the molecular chromophoric subunits not only allows to tune the optical properties of the FONs but also is effective in tuning their surface properties and colloidal stability [12,13]. Among various criteria, the structural (and colloidal stability of FONs is of crucial importance for use as fluorescent nanoprobes for bioimaging. In addition, the FONs brightness is of major importance for sensitive detection and the access to various colors is critical for multicolor detection imaging [14].

Recently, we have shown that “push-pull” dipolar chromophores (D-π-A), built from a *bis*-thiophene π-conjugated system and having a triphenylamine donor (D) moiety could indeed lead to FONs showing improved colloidal stability as a result of the replacement of a single thiophene by a *bis*-thiophene unit [15,16]. Following this route, we herein investigate the effect of replacing the *bis*-thiophene π-conjugated system by a fused analogue, i.e., the thieno[3,2-*b*]thiophene moiety. This modification was meant both to tune the photophysical properties of the chromophoric push-pull subunit and to investigate the influence on the structural and colloidal properties of resulting nanoparticles. In particular, the increased rigidity and planarity of the π-connector is expected to have a favorable effect on the fluorescence properties of the chromophores while it should also influence interchromophoric interactions and self-organization of dyes subunits within FONs.

## 2. Results and Discussion

### 2.1. Design

**Scheme 1:**
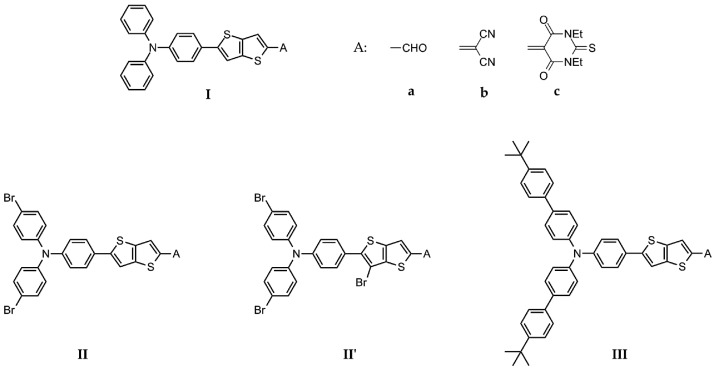
Series of push-pull derivatives **I**–**IIIa**–**c** built from a thieno[3,2-*b*]thiophene π-connector.

In this context, we have prepared and investigated four series of push-pull derivatives bearing different donor and acceptor end-groups (Scheme 1). As a donor, we used the triphenylamino moiety (series **I**), which is well known to be a good electron-donor unit widely used in the design of dipolar and octupolar chromophores showing large two-photon absorption properties [13,17,18,19] (which is essential to get high contrast two-photon imaging). In addition, thanks to its three-dimensional propeller-shape, it acts as a steric impediment preventing perfect anti-parallel stacking of dipolar chromophores upon aggregation, which would otherwise lead to fluorescence quenching. Moreover, we have investigated the effect of the introduction of bromine (series **II**) or *tert*-butylphenyl (series **III**) substituent at the available para positions of the two terminal phenyl groups. This was aimed at both tuning the photophysical properties (by slight modulations of the donating strength) and modulating the colloidal and structural of the subsequent FONs by controlling interchromophoric interactions [12]. Concerning the acceptor moiety, three different acceptors of increasing electron-withdrawing strength have been used: formyl (CHO), dicyanovinyl (DCV) and 1,3-diethyl-2-thiobarbiturate (DETB). The purpose of the variation of the acceptor end-groups was mainly to tune both the linear (absorption and fluorescence) and the non-linear optical (in particular two-photon absorption) properties of the chromophores by playing on their polarization (i.e., on the magnitude of its ground-state dipole moment) [20]. In addition, we also investigated the influence of the presence of a bromine substituent on the thieno[3,2-*b*]thiophene π-connector on both the photophysical properties of the chromophores and on the properties of their FONs (series **II’**) in water.

### 2.2. Synthesis of the Chromophores

**Scheme 2:**
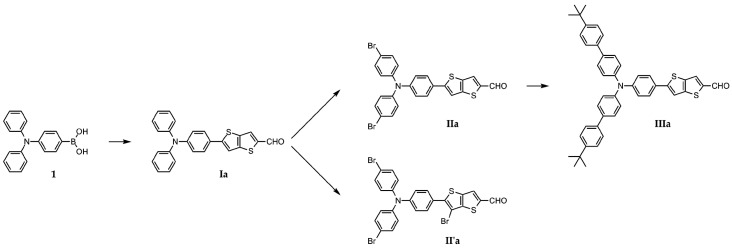
Synthetic route for the preparation of push-pull derivatives **I**–**IIIa**.

The synthetic strategy adopted consists in a combined linear and divergent approach. The first part allows the preparation of push-pull derivatives bearing CHO as the acceptor moiety (derivatives **I**–**IIIa**), as depicted in Scheme 2.

**Scheme 3:**
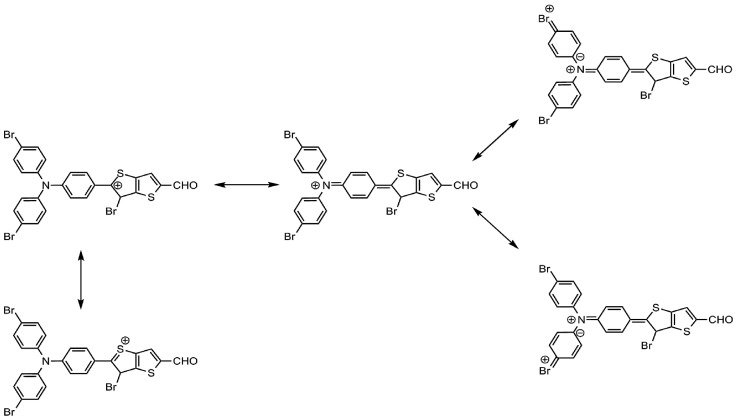
Stabilization of the Wheland intermediate involved in the bromination of the thieno[3,2-*b*]thiophene moiety in β position.

Compound **Ia** represents the key-point of the implemented approach. **Ia** was prepared with good yield (76%) via Suzuki cross coupling between commercially available reagent **1** and 5-bromothieno[3,2-*b*]thiophene-2-carbaldehyde, adapting standard protocols reported in literature for similar 2,2’ bithiophene compounds [16]. A first attempt of direct bromination of **Ia** in order to get **IIa** was done by using 2.1 eq of NBS and a similar protocol as in our previous work [15]. The reaction was monitored by TLC, showing first an intense stain and a weak one close in Rf. Sequential addition of small amounts of NBS induces an inversion of the intensity between the two stains, suggesting that the major compound is a product of extra bromination. After work-up and purification by column chromatography, NMR characterization revealed the formation of **IIa** and **II’a**. The formation of **II’a** can be ascribed to the activation of the β position of the thiophene ring linked to donor moiety (Scheme 3) towards electrophilic substitution. In contrast, the bromination of the β position on the other thiophene ring is prevented due to the electron-withdrawing effect of the CHO substituent on the α position.

Based upon this analysis, optimization of the reaction conditions for the synthesis of **IIa** via bromination of **Ia** was achieved by taking care to not exceed 2.0 eq of NBS. This led to the target molecule **IIa** with high yield (88%). Furthermore, taking advantage of the aforementioned experimental data, we performed tris-bromination of **Ia** using 3.3 eq of NBS, exploiting the reactivity of the active thiophene ring, getting **II**’**a** with high yield too (89%).

**Scheme 4:**
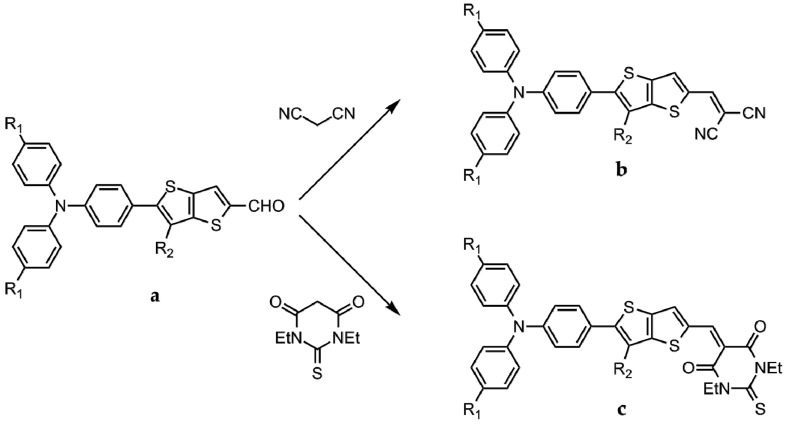
Preparation of push-pull derivatives **I**–**IIIb** and **I**–**IIIc** from compounds **I**–**IIIa** by Knoevenagel reactions.

Derivatives **I**–**IIIa** were then used as precursors for compounds of other series in a divergent fashion by means of Knoevenagel condensations, with malononitrile and 1,3-diethyl-2-thiobarbituric acid (Scheme 4). The experimental conditions of Knoevenagel reactions (in particular nature of the solvent) have been adapted to warrant solubility of the starting material while allowing in situ precipitation of the final product. This ensures displacement of the reaction equilibrium and allows easy collection of the pure product by simple filtration (few exceptions are observed for condensation with malononitrile). These experimental conditions, depicted in Table 1, lead to both high yields and purity degrees.

### 2.3. Photophysical Properties of the New Dyes in Solution in Organic Solvents

#### 2.3.1. One-photon Photophysical Properties of Push-pull Derivatives **I**–**IIIa**–**c** in Solution

The photophysical properties of these new dyes were first investigated in chloroform (CHCl_3_) solutions. The experimental data are summarized in Table 2.

##### Absorption

**Figure 1:**
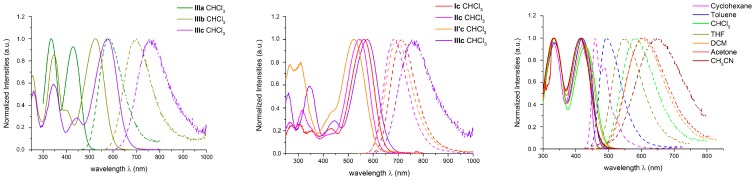
(**left**) Absorption (solid line) and emission (dashed line) spectra of derivatives of series **IIIa**–**c** in CHCl_3_; (**middle**) Absorption and emission spectra of derivatives **I**–**IIIc** in CHCl_3_; (**right**) Solvatochromic behavior of dye **IIIa** in organic solvents of increasing polarities.

All dyes show an intense low-energy absorption band located in the near UV or visible region depending on the dipolar strength. This absorption band characterized by a large molar extinction coefficient (ranging from 2.9 up to 6.8 × 10^4^ M^−1^·cm^−1^) can be ascribed to an intramolecular charge transfer transition (ICT). As illustrated in Figure 1a for series **IIIa**–**c**, increasing the strength of the acceptor end-group (CHO→DCV→DETB) induces a marked bathochromic shift associated with a large hyperchromic effect. Such trend, which is also observed for series **Ia**–**c**, **IIa**–**c** and **II’a**–**c** (see Table 2) is consistent with increasing polarization (i.e., increased contribution of the full ICT, i.e., zwitterionic-mesomeric form to the description of the ground state electronic structure) and larger ground state dipole due to increasing electron-withdrawing strength of the acceptor end-group, thus shifting the electronic structures towards more “cyanine like” structures [20,21].

The strength of the donating end-group parallels the effect of the acceptor end-groups: the presence of a bromine substituent induces a hypsochromic shift (in relation with its inductive effect) while *tert-*butylphenyl induces a bathochromic shift (in relation with its electron-releasing mesomeric effect) as illustrated in Figure 1b for series **I**–**IIIc**. Such trend is also observed for **I**–**IIIa** and **I**–**IIIb** as noted from Table 2. The presence of the bromine substituent on the thieno[3,2-*b*]thiophene π-connector induces both a hypsochromic and hypochromic shift of the low-energy absorption band, which can be related to its inductive electron-withdrawing behavior, that reduces the push-pull strength and the polarization of the dye.

##### Fluorescence

All compounds show fluorescence properties, the effect of the acceptor strength parallels the trend observed with absorption properties: a marked bathochromic shift is observed with increasing electron-withdrawing character as seen from Figure 1a for series **IIIa**–**c** and also noted for series **Ia**–**c**, **IIa**–**c** and **IIIa**–**c** (Table 2). As expected, this large red shift leads to a decrease of the fluorescence quantum yield in relation with both decreasing radiative decay rate [22] and increasing non-radiative decay rate (Table 1). The fluorescence lifetimes are also found to decrease as a result of the marked increase of the non-radiative decay rate. As a result, aldehydes derivatives are bright green to yellow emitters (with fluorescence quantum yield ranging between 0.75 and 0.85), while DCV derivatives are bright red emitters (with fluorescence quantum yield varying between 0.25 and 0.50) and DETB derivatives are far red to NIR emitters (with fluorescence quantum yield varying between 0.05 and 0.40). Interestingly, we observe that the Stokes shift values are decreasing upon increasing the acceptor strength, which is consistent with the electronic structure of the dyes shifting closer to the cyanine limit.

The effect of the substituents on the donating end-group also parallels the trend observed in absorption. As observed from Figure 1b for series **I**–**IIIc** (and from Table 2 for series **I**–**IIIa** and series **I**–**IIIb**), the bromine substituents induce a hypsochromic shift of the emission band, while the *tert-*butylphenyl ones induce a bathochromic shift. The latter also induce a decrease of the fluorescence quantum yield due to a lower radiative decay rate (k_r_), in relation with the red-shifted emission [22] and larger non-radiative decay rate (k_nr_). We note that in the case of DCV and DETB derivatives (i.e., **IIIb** and **IIIc**), both the fluorescence quantum yields and the lifetimes show a marked decrease in relation with much larger non-radiative decay rates (increased by a factor 2 for DCV derivatives and by a factor 5 for DETB derivatives). In contrast, the aldehyde derivatives do not show such behavior which suggests that additional non-radiative decay processes—such as photo-induced electron-transfer—are operative in the case of the push-pull derivatives bearing very strong donor and acceptor end-groups. In addition, the bromine substituent also seems to promote efficient non-radiative decay processes (most probably intersystem crossing due to spin-orbit coupling effect) in the case of DCV and DETB derivatives (i.e., **IIb** and **IIc**), leading to smaller fluorescence quantum yields and shorter lifetimes (Table 2).

In that regard, the effect of the additional bromine atom as a substituent on the thieno[3,2-*b*]thiophene π-bridge is of particular interest. Contrary to the absorption band, the emission spectra is slightly red-shifted, leading to larger Stokes shift values in the case of derivatives **II’** compared to homologous derivatives **II**. Yet, compounds bearing the stronger end-groups (**II’b** and **II’c**) show larger fluorescence quantum yield and longer fluorescence lifetimes than their homologues **IIb** and **IIc**, indicating that the presence of the bromine on the π-connector hinders the competing non-radiative decay process (such as photo-induced electron transfer) that is deleterious to fluorescence (see Table 2).

##### Effect of the Environment

The effect of the environment surrounding single dye in solution was also investigated by monitoring the variation in both absorption and emission features by tuning the solvent polarity. It can be observed in Figure 1c (see also Supplementary Materials) that the dyes display a marked positive solvatochromism in emission, which is consistent with an ICT transition associated with a large increase of the dipolar moment upon excitation. In addition, a marked decrease of the fluorescence quantum yield is observed in polar solvents in connection with red-shifted emission and augmented photo-induced electron-transfer. It is important to note that the derivatives of series **b** and **c** (i.e., having DCV and DETB acceptor end-groups) are found to hydrolyze in polar hygroscopic solvents such as acetonitrile and DMSO. These polar solvents, which contain traces of water favor retro-Knoevenagel reactions (vide infra). This reaction was found to be even faster for derivatives **I**–**IIIc** than for derivatives **I**–**IIIb**. Dye **IIIb** starts to show decomposition after one day while dye **IIIc** decomposes after only few minutes in diluted solution in DMSO.

#### 2.3.2. Two-Photon Absorption Properties of Push-pull Derivatives **I**–**IIIa**–**c** in Solution in Chloroform

Thanks to their luminescence properties, we were able to determine the 2PA cross section (σ_2_) of the chromophores by investigating the two-photon excited fluorescence (2PEF) in CHCl_3_ solution. The two-photon absorption spectra (2PA) were recorded in a spectral range from 700 nm to 1150 nm (depending on the shift of the emission band of the chromophore) using the TPEF technique following the well-established method described by Webb and coworkers [23]. This technique has the advantage to yield reliable data for fluorescent compounds by checking the quadraticity of the induced emission signal on the excitation intensity.

**Figure 2:**
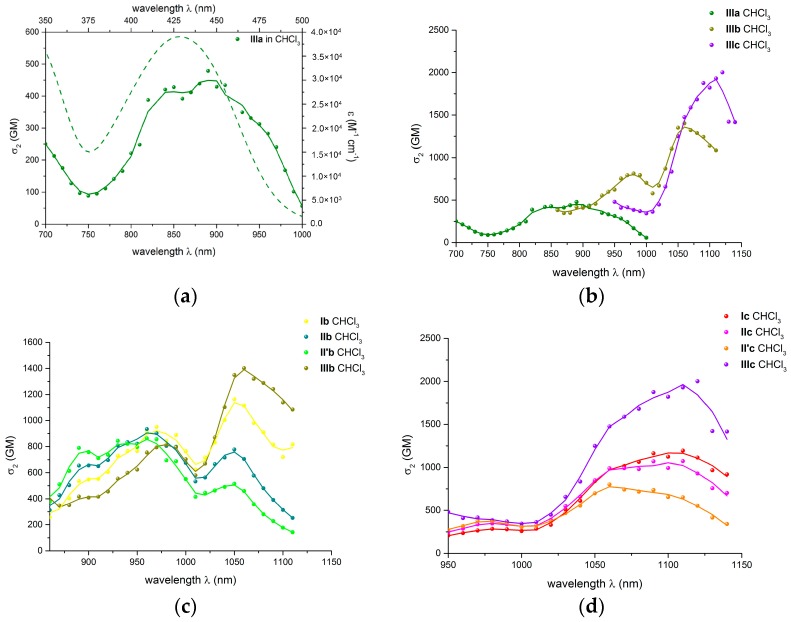
(**a**) 1PA (dashed line) and 2PA (colored points and solid line) spectra measured for the chromophore **IIIa** in CHCl_3_; (**b**) Comparison between two-photon absorption spectra for the dyes **IIIa**–**c** in CHCl_3_; (**c**) Comparison between the 2PA spectra of derivatives **I**–**IIIb** in CHCl_3_; (**d**) Comparison between the 2PA spectra of derivatives **I**–**IIIc** in CHCl_3_.

All chromophores display a broad 2PA band, as shown in Figure 2a for compound **IIIa.** We note that the maximum 2PA wavelength (λ_max_^2PA^) is approximately located at about twice the wavelength of the one-photon absorption band (1PA). This indicates that the low-energy excited state associated with the ICT transition is both allowed upon one-photon and two-photon excitation, as expected for push-pull chromophores which lack a center of symmetry [24]. As illustrated in Figure 2b for series **IIIa**–**c** (and displayed in Table 2 for series **Ia**–**c**, **IIa**–**c** and **II’a**–**c**), increasing the electron-withdrawing group strength induces a marked red-shift of the 2PA band in agreement with the trend observed for the ICT band. The maximum 2PA cross-section (σ_2_^max^) values are also found to increase dramatically on going from **a** to **b** derivatives then more softly on going from **b** to **c** derivatives (most probably in relation with a degree of ICT in the ground state close to the optimum polarization for maximum 2PA response in the case of derivatives having DCV or DETB acceptor end-groups [21]).

Similarly, increasing the strength of the electron-releasing moiety leads to a slight red shift of the 2PA band and increase of the maximum 2PA cross-section, as illustrated in Figure 2c,d for series **I**–**IIIb** and **I**–**IIIc**. While the bromine substituents lead to a slight decrease of the 2PA response (in relation with a decrease of the donating strength) except for derivatives **Ia**–**IIa**, the *tert-*butylphenyl induces a marked increase of the 2PA response (see also Table 2 for series **I**–**IIIa**). Finally, in all cases the introduction of an additional bromine atom as a substituent on the thieno[3,2-*b*]thiophene π-conjugated bridge always leads to a decrease of the 2PA response (see Figure 2c,d and Table 2).

### 2.4. Preparation and Morphological Characterization of the FONs in Water

Several methods have been reported for the preparation of molecular based organic nanoparticles ranging from laser ablation [25,26,27] to vapor [28] or electrochemical [29] deposition. The simplest and widely used in the scientific community is the reprecipitation method published firstly by Nakanishi and coworkers [25,30]. To prepare the water-soluble FONs, we used the nanoprecipitation process by injecting a small volume of mM solution of the dye in THF in a large volume of milliQ water in which the compound is not soluble. The abrupt change of the environment induces a fast nanoaggregation of the dyes in pseudo-amorphous nanoparticles as indicated by absorption spectra.

**Figure 3:**
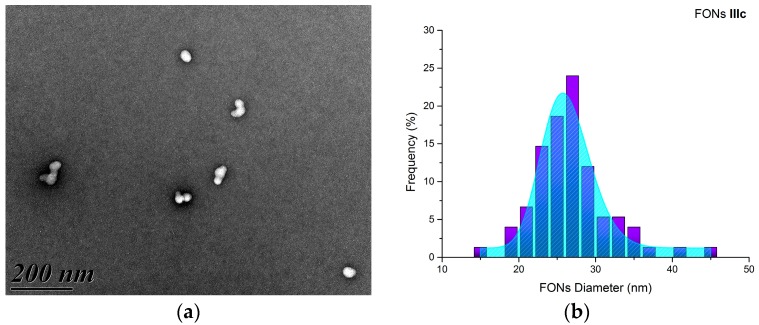
(**a**) TEM image of FONs **IIIc**; (**b**) Size distribution and Log Normal fitting of FONs **IIIc**.

The morphological characterization was carried out using transmission electron microscopy (TEM). The nanoparticles obtained are spherical and show slight polydispersity as illustrated in Figure 3 for FONs made from **IIIc**. The diameters of the FONs are typically ranging from 21 nm to 43 nm with average diameter of 32 nm. From the TEM diameter values, we could estimate the average number of chromophores within a single nanoparticle. As shown in the Table 3, the numbers N of dyes per single nanoparticle amount to about a few tens of thousands, varying from 0.6 × 10^4^ for FONs made from dyes **Ib** and **II’c**, up to 5.7 × 10^4^ for FONs made from dye **Ia**, which gives the biggest nanoparticles.

All FONs made from push-pull derivatives **I**–**IIIa**–**c** display a highly negative surface potential, down to −85 mV for FONs made from dye **IIa**. The highly negative surface of the FONs suggests that the dipolar dyes show specific arrangement and short distance order near the surface [12], with most probably electron-withdrawing groups (which bear O or N atoms having lone pairs and liable to be H-bond acceptors) pointing towards the water interface. These highly negative surface potential values are favorable features in view of the colloidal stability.

### 2.5. Photophysical Properties of Fluorescent Organic Nanoparticles in Water

The photophysical properties of the FONs made from dyes **I**–**IIIa**–**c** in water are collected in Table 4. All FONs show an intense absorption band in the visible region and retain some fluorescence in water although they show definite aggregation-caused quenching (ACQ).

#### 2.5.1. One-photon Photophysical Properties of FONs in Water

**Figure 4:**
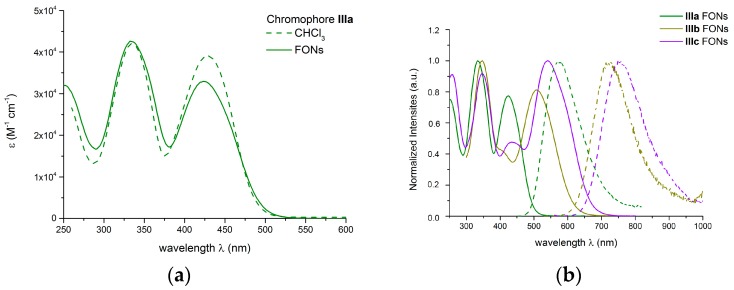
(**a**) Comparison of the absorption spectra of dye **IIIa** as isolated chromophore dissolved in CHCl_3_ and as chromophoric subunits in FONs; (**b**) Comparison of absorption (solid line) and emission (dashed line) spectra of FONS made from dyes **IIIa**, **IIIb** and **IIIc** in water.

Alike dyes in solution, the FONs show an intense low-energy absorption band in the visible region, whose position ranges from the violet to the green spectral region depending on the strength of the donor and acceptor end-groups (Table 4). As observed from Table 4, molecular confinement of the push-pull dyes within FONs induces a blue-shift as well as a slight hypochromic effect (except for dyes **IIb** and **IIIb**) as compared to dyes in solution. In addition as illustrated in Figure 4a for dye **IIIa**, a slight broadening of the ICT band is observed (see also SI). This behavior could be ascribed to excitonic coupling favored by the close proximity of the dyes within FONs. We note that this effect is much less pronounced for push-pull derivatives **I**–**IIIa** bearing the weaker acceptor end-group (i.e., CHO), in relation with their smaller transition dipole moment. In addition, in all cases, we observe that the blue-shift is smaller for derivatives that have donor end-groups which bear bulky substituents (either bromine or *tert-*butylphenyl). The steric hindrance generated by these substituents increases the distance between chromophoric subunits and thus reduces excitonic coupling. This effect is even more pronounced for derivatives **II’**, in which the presence of the additional bromine substituent on the thienothiophene π-bridge further increases spacing between dyes subunits (Table 4).

The nature of the end-groups influence the absorption and emission spectra in a similar way as was the case for the dyes dissolved in chloroform. Increasing the strength of the electron-withdrawing moiety, from CHO to DCV and DETB, induces a marked bathochromic shift of both absorption and emission spectra as observed from Table 4 and illustrated in Figure 4b for derivatives **IIIa**–**c.** Similarly, decreasing the strength of the electron-donor (**I**→**II**) induces a hypsochromic shift of both absorption and emission (Table 4) as was observed in organic solution. In contrast, increasing the strength of the electron-donor (**I**→**III**) always leads to a bathochromic shift of the absorption spectra but induces a bathochromic shift of the emission spectra only in the case of derivatives bearing the weakest electron acceptor (**Ia**→**IIIa**). At opposite, derivatives FONs made from dyes **IIIb** and **IIIc** show blue-shifted emission as compared to FONs made from dyes **Ib** and **Ic**. Finally the presence of the extra bromine onto the π-bridge leads to very slight blue-shift of the low energy (ICT) absorption band and slightly blue-shifted emission contrarily to what was observed in solution (Table 1).

As indicated above, the FONs show significantly lowered fluorescence as compared to the dyes dissolved in a low polarity solvent. This ACQ phenomenon can be related to both environmental and interchromophoric interactions effects. We note that whereas FONs made from derivatives **I**–**IIIa** show emission spectra which reveal a medium polarity environment (typically varying from that of chloroform to that of DCM based on the solvatochromism of the dyes in solution, see Supplementary Materials) FONs made from dyes **I**–**IIb** and **I**–**IIc** display fluorescence emission which reveal a polar environment (typically similar to acetone). As the dipolar moment of the push-pull dyes subunits increases on going from derivatives bearing CHO to DCV and DETB electron-withdrawing end-groups, the push-pull dyes create upon molecular confinement a local environment of increasing polarity (thus leading to lower fluorescence quantum yield). In addition, due to close molecular confinement, intermolecular photo-induced electron transfer (i.e., intermolecular charge transfer) is rendered possible within FONs and favored by a polar environment. Such phenomenon is particularly active in derivatives of series **I**–**IIIc** which bear the strongest electron-donating and electron-withdrawing end-groups (with fluorescent quantum yield values lower than 1%) and also in derivatives of series **I**–**IIIb** (with fluorescent quantum yield values in between 1% and 2%). Derivatives of series **I**–**IIa** show slightly larger fluorescence quantum yields (5%–6%) in agreement with their lower polarity and less strong end-groups. In addition, it is interesting to note that derivatives **III** and **II’** show both the largest fluorescence quantum yield in each family as well as emission corresponding to the lowest polarity environment. This can be explained by the presence of the bulky groups (bromine and *tert*-butylphenyl substituents) which increases the distance between the chromophoric subunits within the FONs and thus both reduce the local field generated by its closes neighbors experienced by the dyes within FONs as well as excitonic coupling.

We observe that all FONs display a biexponential fluorescence decay: both a shorter and a longer lifetimes are obtained as compared to the dyes dissolved in chloroform (see Table 2 and Table 4). These two different lifetimes point to dyes experiencing distinct environments. The longer lifetime may be attributed to the dyes located at the core of the nanoparticles (in relation with the red-shifted emission as compared to chloroform and reduced vibrational decay rate). The shortest lifetime might be attributed to dyes located closer to the surface where efficient non-radiative decay processes (i.e. intermolecular electron-transfer and vibrational deactivation involving H-bonding to the water molecules located at the interface) are facilitated. We note that the contribution of the shortest lifetime is clearly dominant in derivatives of series **I**–**IIIc** explaining the very low fluorescence quantum yield. This can be ascribed to efficient photo-induced intermolecular electron transfer promoted by the water proximity.

Interestingly, although all FONs undergo ACQ phenomenon, they still retain very large brightness (varying from 10^6^ M^−1^·cm^−1^ to 10^8^ M^−1^·cm^−1^), thanks to their large absorption coefficient (Table 5). In particular, green to yellow emitting FONs made from derivatives **Ia**, **IIa** and **IIIa** and deep-red to NIR-emitting FONs made from derivatives **IIIb** show huge brightness (0.4–1.0 × 10^8^ M^−1^·cm^−1^). NIR-emitting FONs made from derivatives **I**–**IIIc** show a one-order of magnitude smaller brightness, in relation with both their very low fluorescence quantum yield and their smaller size.

#### 2.5.2. Two-Photon Absorption Properties of the FONs in Water

**Figure 5:**
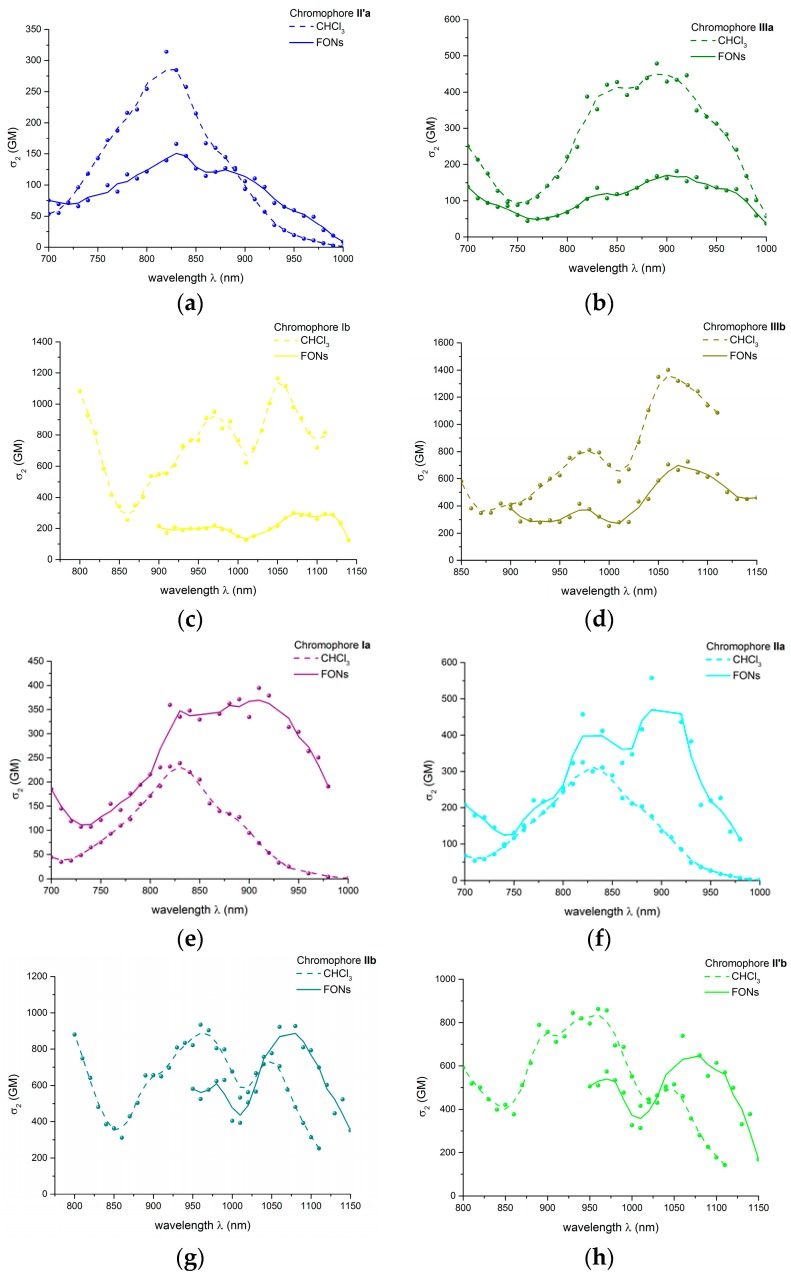
Comparison of the 2PA spectra of the dye dissolved in CHCl_3_ and as subunits of FONs in water: (**a**) **II’a**; (**b**) **IIIa**; (**c**) **Ib**; (**d**) **IIIb**; (**e**) **Ia**; (**f**) **IIa**; (**g**) **IIb**; (**h**) **II’b**.

Thanks to their emissive properties, the 2PA properties of FONs made from the dyes of series **I**–**IIIa** and **I**–**IIIb** could be determined by investigating their two-photon induced fluorescence properties in water. Yet, NIR-emitting FONs made from dyes of series **I**–**IIIc** showed too low fluorescence quantum yield to allow reliable 2PEF measurements. The experimental data are shown in Figure 5 and summarized in Table 4 where the maximum two-photon absorption cross-section values (σ_2_^max^) per dye subunit within FONS dispersed in water are collected.

As was observed in the case of the dyes dissolved in an organic solvents, the maximum wavelength related to the 2PA process is similar or slightly red-shifted compared with twice the value of λ_abs_^max^, suggesting that the corresponding excited state is both one-and two-photon allowed (Table 4). On the other hand, the molecular confinement of the dyes within FONs strongly affects their 2PA response (Table 5). Indeed, dipolar interactions are known to strongly influence the 2PA response of polar and polarizable chromophores [31]. Due to molecular confinement, such effects are expected to be quite strong within FONs made from dipolar dyes, depending both on the relative orientation and distance of the dyes and on their polarity/polarizability [32,33].

Actually, we observe that the dipolar interactions lead to a *major decrease* of the 2PA response per chromophoric subunit in the case of FONs made from dyes **II’a** and **IIIa** (by a factor of 2–3; see Figure 5a,b) as well as for FONs made of dyes **Ib** and **IIIb** (by a factor of 2–4, see Figure 5c,d). In contrast, the interchromophoric dipolar interactions lead to an *increase* of the 2PA response per chromophoric subunits in the case of FONs made from dyes **Ia** and **IIa** (see Figure 5e,f). Such effect has also been reported recently in FONs made from different dyes subunits (i.e., articulated dipoles) investigated in a previous work [34]. Finally, in the case of FONs made from dyes **IIb** and **II’b**, the interactions lead to clear modification of the vibronic substructure as illustrated in Figure 5g,h. As a result, the positions of the 2PA maxima are reversed while the 2PA maximum cross-sections are only slightly affected.

**Figure 6:**
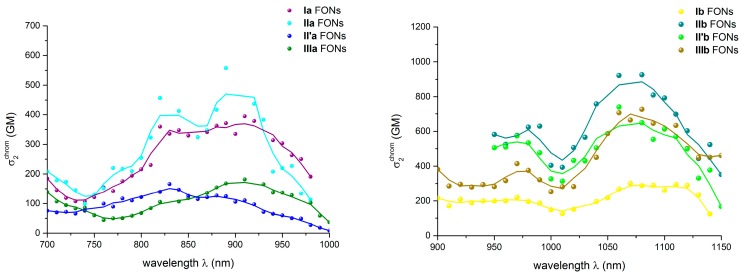
Comparison of the 2PA spectra of dyes for FONs of series **I**–**IIIa** (**left**) and **I**–**IIIb** (**right**).

As expected, the effect of interchromophoric interactions on the 2PA response of the dyes subunits within FONs is found to be strongly modulated by the presence of the bulky substituents as those influence the relative positioning (and closest distance) of the dye subunits in FONs. As result, the 2PA responses of the different FONs are found to follow different ordering as those of dyes in solution. In particular, the strongest donating end-groups (i.e., having *tert-*butylphenyl substituents) do not show the largest 2PA response as illustrated in Figure 6a,b for derivatives of series **I**–**IIIa** and **I**–**IIIb**. In contrast, the bromine substituents (i.e., FONs made from dyes **II**) lead to the largest 2PA response in all cases, indicating that it favors the more favorable relative orientation of dyes subunits within FONs to promote 2PA enhancement.

**Figure 7:**
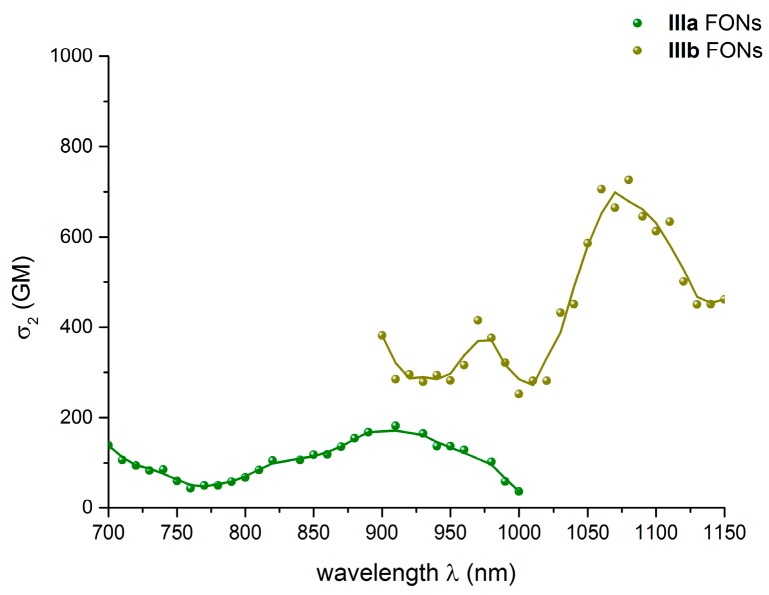
Comparison of the 2PA spectra of dyes as FONs subunits for derivatives **IIIa** and **IIIb.**

On the other hand, increasing the strength of the electron-withdrawing end-groups induces both a strong bathochromic shift and a marked enhancement of the two-photon absorption cross section (Figure 7), as was observed for the dyes in organic solution.

As a result of the combined effect of molecular confinement on the 2PA response and on the fluorescence quantum yield, a selection of tunable FONS showing giant two-photon brightness can be identified. Indeed, green-emitting FONs made from the dye **IIa**, and yellow-emitting FONs made from the dye **Ia** show 2P brightness of about 10^6^ GM surpassing all other types of nanoparticles (including quantum dots) while deep red-NIR emitting FONs made from dyes **IIb** and **IIIb** exhibit 2P brightness of about 0.5 × 10^6^ GM (Table 5) [35,36].

### 2.6. FONs Stability

**Figure 8:**
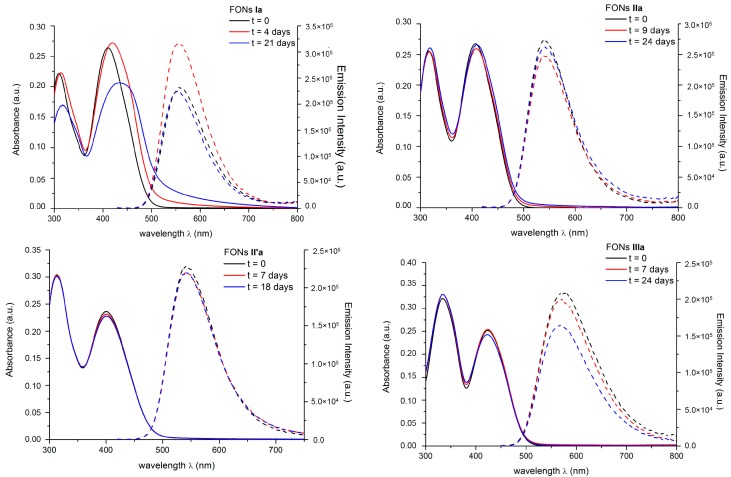
Monitoring of the evolution of the absorption and emission features of FONs made from dyes **I**–**IIIa** in water over time.

In order to investigate the structural and colloidal stability of FONs made from dyes **I**–**IIIa**–**c**, we have monitored the evolution of their absorption and emission properties overtime. The colloidal stability is not the only goal of bottom-up engineering of FONs for bioimaging purposes. In fact, in many cases, where the colloidal stability is found to be suitable (i.e., no change of size and morphology over time), the luminescence properties of FONs are found to decrease markedly over time [32], in relation with slow surface rearrangements processes. These characteristics are related to the structure of chromophoric subunits and their interactions within FONs and thus are expected to be strongly influenced by the structure of the molecular subunit. This is illustrated in Figure 8.

FONs made from dye **Ia** clearly show an increase in size over time, with noticeable flattening and broadening of the ICT absorption band as well as onset of scattering. In contrast, the presence of bulky substituents such as *tert*-butylphenyl groups or bromine atoms, as substituents on the donor moiety and/or along the π-bridge, significantly improves the colloidal stability of resulting FONs whose absorption characteristics remain unchanged even after a few weeks. Interestingly, we observe that fluorescence properties are also totally preserved over time for FONs made from dyes **IIa** and **II’a**. Hence, the bromine substituents are found to be even better appendices to improve the structural stability of the surface than *tert*-butylphenyl substituents.

FONs made from dyes bearing stronger acceptor end-groups are found to show slightly lower colloidal stability (see Supplementary Materials). FONs made from dyes **II’b** and **IIIb** (i.e., bearing *tert*-butylphenyl substituents or three bromine substituents) are found to show the best colloidal stability of series **I**–**IIb** while FONs made from dyes **Ic** and **IIIc** are found to be the most stable. The fluorescence properties of FONs made from derivatives **II’**–**IIIb** are well maintained over time while those of FONs made from derivatives **Ic** and **IIIc** slowly decrease, indicating a less endoergonic exchange energy for these dyes [12].

**Figure 9:**
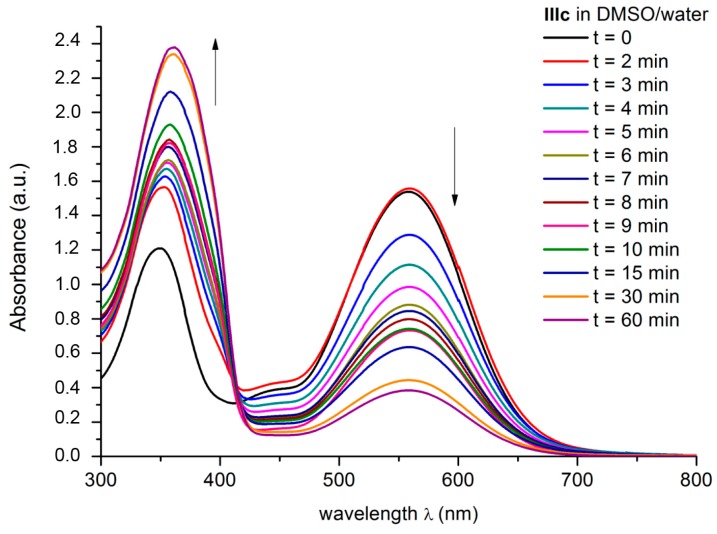
Monitoring of the evolution of the absorption spectra of dye **IIIc** in DMSO after addition of 2% of water.

**Figure 10:**
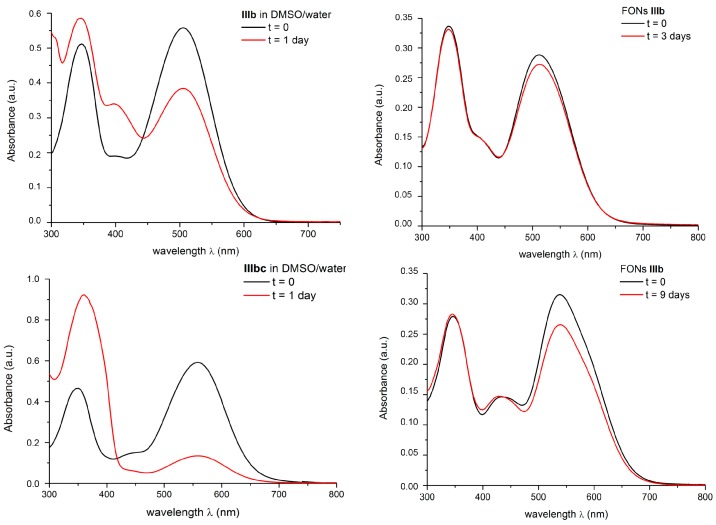
Chemical stabilization of dyes **IIIb** and **IIIc** within FONs.

Strikingly, whereas dyes from series **I**–**IIIb** and **I**–**IIIc** are found to be unstable over time in polar solvents in the presence of traces of water, as they undergo a retro-Knoevenagel reaction (Figure 9), their chemical stability is observed to be greatly improved upon molecular confinement within FONs (Figure 10). This clearly demonstrates the chemical stability of the FONs although the dyes subunits are not stable as single molecules in organic solvent in the presence of water. The dyes are thus fully chemically protected within FONs and the structural stability of the nano-aggregate is large enough to prevent their exchange with bulk water.

### 2.7. Core-Shell Nanoparticles

The photophysical properties of the FONs make them of major interest as bright contrast agents for biological optical imaging in cells with visible excitation as well as tissue imaging with NIR excitation (via 2P excitation). However, the low fluorescence quantum yield of the NIR emitting FONs (series **I**–**IIIc**) which are of major promise for in vivo imaging is responsible for a marked reduction of their one and two-photon brightness. We thus proceeded to circumvent this shortcoming. Our working hypothesis was that the marked decrease of the luminescence of the FONs made from NIR emitting dyes predominantly originated from efficient deactivation processes mediated by the FONs/water interface (vibrational deactivation mediated by H-bonded water molecules or intramolecular charge transfer favored by water). 

We envisaged two different approaches. The first one is based on the replacement of the water shell bound at the surface of the FONs by cationic polymers that interact *via* electrostatic interactions. We recently showed this route to be an efficient strategy for increasing the fluorescence efficiency of NIR emitting FONs made from quadrupolar dyes [37]. However, with the aim of using the FONs as in vivo nano-labels, we privileged an alternative approach, which does not require additives. We thus aimed at designing synergic fluorescent molecular-based core-shell nanoparticles. We have shown recently that core-shell FONs can be obtained using a sequential nanoprecipitation protocol by using well-chosen complementary dipolar dyes. These core-shell FONs promote highly efficient shell-to-core energy transfer, as well as enhancement (by a factor of three) of the fluorescence quantum yield of the emitting core excited via a FRET process [38,39]. For the design of synergic core-shell FONs, we selected a pair of optimized complementary push-pull dyes. The emission band of the donor dye **IIa** shows perfect overlap with the absorption band of the acceptor dye **IIIc** (see Table 4) while FONs made from **IIIc** show NIR emission (though with very low fluorescence quantum yield) and FONs made from **IIa** combine giant 2PA response (Table 4) and very good colloidal stability (vide supra).

**Figure 11:**
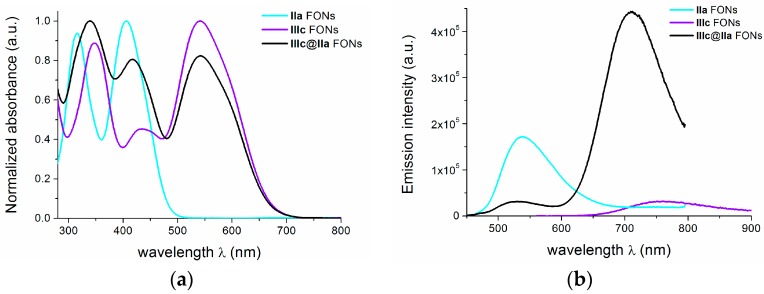
(**a**) Normalized absorption spectra of FONs **IIa**, **IIIc** and **IIIc@IIa** in water; (**b**) Emission spectra of FONs **IIa** (λ_exc_ = 406 nm), **IIIc** (λ_exc_ = 542 nm) and **IIIc@IIa** (λ_exc_ = 406 nm).

The preparation of the core-shell FONs was achieved by performing a dropwise addition of a solution of the donor **IIa** (1 mM in THF) to a freshly prepared aqueous solution of the FONs made from the acceptor (FONs **IIIc**). After a few minutes stirring, the absorption and emission spectra of the solution validate the formation of core-shell FONs. The absorption spectrum of the resulting clear red solution show the characteristic bands of both dyes **IIa** and **IIIc** (Figure 11a) while the fluorescence emission spectrum of the solution excited at maximum absorption wavelength of the donor (**IIa**) reveals an intense NIR emission, instead of the green one expected for FONs made from pure donor **IIa** (Figure 11b). In addition, a marked increase of the fluorescence intensity associated with a slight hypsochromic shift of the maximum of fluorescence is observed as compared to that of starting FONs made from pure acceptor **IIIc**. This singular behavior is the specific mark of the formation of binary core-shell FONs **IIIc@IIa**.

The slight hypsochromic shift of the fluorescence emission spectrum of FONs **IIIc@IIa** as compared to that of pure FONs made from **IIIc** indicates that the polarity surrounding the acceptor emitters (i.e., dyes **IIIc** located close to the interface with the shell) is lower than the one experienced by dye subunits in FONs made from dye **IIIc** only. This is consistent with the less polar environment experienced by dyes **IIIc** located close to the interface and excited by FRET. The FRET process is confirmed by the variation of the fluorescence lifetimes. As shown in Table 6, the lifetimes of the green emission of the donor (dye **IIa**) in FONs **IIIc@IIa** are significantly reduced as compared to that of FONs made from pure dye **IIa**.

The large amplification of the core NIR emitter fluorescence (by a factor of 20, Table 6, i.e., much larger than the one we previously reported in ref. [38]) can be ascribed both to an increase of the radiative decay rate (mediated by the electric field generated at the interface between the core and shell of FONs **IIIc@IIa**) and a decrease of non-radiative processes (due to the protection from water). The variation of the fluorescence lifetimes of the core emitter are of interest in that respect. As shown in Table 6, the lifetimes of the NIR emission of acceptor **IIIc** are increased in FONs **IIIc@IIa** upon excitation of the donor (dye **IIa)** as compared to that FONs made from pure **IIIc**. The shorter lifetime is increased by a factor 2, which is consistent with a reduction of non-radiative decay processes mediated by the interface (in agreement with the protection from water proximity). The longer lifetime is also lengthened (by about 50%) while its contribution is found to increase.

As such, these core-shell FON_S_ offer major promise for bioimaging as they combine the giant 2P absorption cross-section of the shell made from **IIa** when excited at 890 nm (15 × 10^6^ GM, see Table 5) and NIR emission from core emitter **IIIc**. This results in FONs allowing both excitation and emission in the NIR region and showing unprecedented 2P brightness (1.5 × 10^6^ GM for excitation at 890 nm and emission at 710 nm). As such, these FONs offer major promises for in vivo imaging.

## 3. Materials and Methods

Commercially available reagent (purchased from Aldrich (L’isle d’Abeau Chesnes, France), Alfa Aesar (Karlsruhe, Germany) and TCI (Zwijndrecht, Belgium)) were used without further purification. Dry solvents were distilled from the appropriate drying reagents immediately before use. All air- or water-sensitive reactions were carried out under argon. Chromatography columns were performed using Fluka silica gel Si 60 (40–63 µm, 230–400 mesh).

### 3.1. Chemical Characterizations

Infrared spectra were measured on a Perkin Elmer Spectrum 100 Optica (Perkin Elmer, Wellesley, MA, USA). ^1^H- and ^13^C-NMR spectra were recorded on a Bruker Avance III 200 spectrometer at 200 MHz and 50 MHz respectively, Bruker Avance I 300 at 300 MHz and 75 MHz respectively, on a Bruker Advance II 400 spectrometer at 400 MHz and 100 MHz respectively, on a Bruker Advance III 600 spectrometer at 600 MHz and 150 MHz respectively (Bruker, Billerica, MA, USA). Shifts (δ) are given in parts per million with respect to solvent residual peak and coupling constants (*J*) are given in Hertz. HRMS (FD) spectra were performed by CESAMO (Bordeaux, France). The measurements were carried out on a TOF mass spectrometer AccuTOFGCv using an FD emitter with an emitter voltage of 10 kV. One to two microliters solution of the compound is deposited on a 13 μm emitter wire. Elemental analyses were carried out by the “Institut de Chimie des Substances Naturelles” (Gif-sur-Yvette, France). Melting points were measured on Stuart SMP 10.DSC measures were carried out on Mettler Toledo DSC 1 STAReSystem (Mettler, Greifensee, Switzerland).

### 3.2. Experimental Part

*5-(4-(Diphenylamino)phenyl)thieno[3,2-b]thiophene-2-carbaldehyde* (**Ia**): To a solution of **1** (1.00 g, 3.450 mmol) in 16.0 mL of anhydrous and degassed toluene/MeOH mixture (1:1), 5-bromothieno[3,2-*b*]thiophene-2-carbaldehyde (0.812 g, 3.280 mmol), K_2_CO_3_ (1.13 g, 8.210 mmol), Pd(dppf)Cl_2_ (134.0 mg, 0.154 mmol) were added and the mixture was stirred overnight at 75 °C. After cooling down to room temperature, the crude was concentrated and few milliliters of CH_2_Cl_2_ were added. The suspension was filtered and plenty rinsed with CH_2_Cl_2_. The filtered was purified by means of chromatography column (petroleum ether:CH_2_Cl_2_ = 6:4) obtaining 1.03 g of **Ia** as a yellow powder (76%).

*5-(4-(Bis(4-bromophenyl)amino)phenyl)thieno[3,2-b]thiophene-2-carbaldehyde* (**IIa**): To a solution of **Ia** (200.0 mg, 0.486 mmol) in 22.0 mL of anhydrous THF cooled at 0 °C, NBS (176.5 mg, 0.991 mmol) was added and the mixture was stirred for 6 h. The solution was slowly warmed to room temperature and stirred for 12 h. 22.0 mL of water were added and extraction with CH_2_Cl_2_ was done. The organic layer was washed with NaHCO_3(sat)_, water, dried over Na_2_SO_4_ and filtered. Evaporation of the solvents gave a yellow powder which is purified by means of chromatography column (CH_2_Cl_2_), obtaining 244.0 mg of **IIa** as a yellow powder (88%). DSC > 204 °C (decomp.); ^1^H-NMR (200 MHz, acetone-*d*_6_): δ 10.01 (s, 1H), 8.28 (s, 1H), 7.84 (s, 1H), 7.72 (d, *J* = 8.6 Hz, 2H), 7.51 (d, *J* = 8.8 Hz, 4H), 7.14 (d, *J* = 8.6 Hz, 2H), 7.09 (d, *J* = 8.8 Hz, 4H); ^13^C-NMR (50 MHz, acetone-*d*_6_): δ 184.3, 152.9, 148.6, 147.3, 147.1, 145.7, 138.8, 133.5, 131.1, 129.4, 128.1, 127.3, 124.7, 116.9, 116.6; ATR (cm^−1^): 1657 ν(C=O); HRMS (FD) calcd for C_25_H_15_Br_2_NOS_2_ *m/z*: 566.8962, found: 566.8935; elemental analysis calcd (%) for C_25_H_15_Br_2_NOS_2_ + 0.04 C_2_H_2_Cl_2_: C 52.51, H 2.65, N 2.45, found: C 52.25, H 2.59, N 2.58.

*5-(4-(Bis(4-bromophenyl)amino)phenyl)-6-bromothieno[3,2-b]thiophene-2-carbaldehyde* (**II’a**): To a solution of **Ia** (30.0 mg, 0.073 mmol) in 3.5 mL of anhydrous THF cooled at 0 °C, NBS (43.0 mg, 0.240 mmol) was added and the mixture was stirred for 0.5 h. The solution was warmed to room temperature and stirred for 72 h. 4.0 mL of water were added and extraction with CH_2_Cl_2_ was done. The organic layer was washed with NaHCO_3(sat)_, water, dried over Na_2_SO_4_ and filtered. Evaporation of the solvents gave a yellow powder which is purified by means of a short pad of silica (CH_2_Cl_2_), obtaining 42.0 mg of **II’a** as a yellow powder (89%). DSC > 240 °C (decomp.); ^1^H-NMR (200.0 MHz, CD_2_Cl_2_): δ 9.96 (s, 1H), 8.00 (s, 1H), 7.64 (d, *J* = 8.7 Hz, 2H), 7.43 (d, *J* = 8.8 Hz, 4H), 7.11 (d, *J* = 8.7 Hz, 2H), 7.03 (d, *J* = 8.8 Hz, 4H); ^13^C-NMR (150 MHz, CD_2_Cl_2_): δ 183.6, 155.7, 148.8, 148.4, 146.3, 146.1, 144.7, 135.6, 133.0, 130.3, 129.9, 126.9, 123.1, 117.0, 100.2; ATR (cm^−1^): 1663 ν(C=O); HRMS (FD) calcd for C_25_H_14_Br_3_NOS_2_ *m/z*: 644.8067, found: 644.8062; elemental analysis calcd (%) for C_25_H_14_Br_3_NOS_2_: C 46.32, H 2.18, N 2.16, found: C 46.41, H 1.84, N 2.09.

*5-(4-(Bis(4'-(tert-butyl)-[1,1'-biphenyl]-4-yl)amino)phenyl)thieno[3,2-b]thiophene-2-carbaldehyde* (**IIIa**): Air was removed from a mixture of **IIa** (250.0 mg, 0.440 mmol), 4-*tert*-butylphenylboronic acid (215.0 mg, 1.208 mmol), Pd(dppf)Cl_2_ (72.0 mg, 0.088 mmol) and K_2_CO_3_ (365.0 mg, 2.641 mmol) in 6.0 mL of toluene/MeOH (1:1) by blowing argon for 30 min. The mixture was warmed to 75 °C for 24 h. The reaction was cooled to room temperature and quenched by addition of water. After extraction with CH_2_Cl_2_, drying over Na_2_SO_4_ and filtering through a short pad of Celite, the crude was concentrated under vacuum and purification was achieved by chromatography column using gradient elution (petroleum ether, petroleum ether:CH_2_Cl_2_ = 75:25, petroleum ether:CH_2_Cl_2_ = 60:40) to yield 211.0 mg of **IIIa** as an orange powder (71%). DSC > 155 °C (decomp.); ^1^H-NMR (300 MHz, DMSO-*d*_6_): δ 9.96 (s, 1H), 8.39 (s, 1H), 7.92 (s, 1H), 7.71 (d, *J* = 8.6 Hz, 2H), 7.65 (d, *J* = 8.5 Hz, 4H), 7.59 (d, *J* = 8.4 Hz, 4H), 7.47 (d, *J* = 8.4 Hz, 4H), 7.20 (d, *J* = 8.5 Hz, 4H), 7.13 (d, *J* = 8.6 Hz, 2H), 1.32 (s, 18H); ^13^C-NMR (75 MHz, CD_2_Cl_2_): δ 183.4, 153.4, 150.6, 148.9, 147.3, 146.5, 144.6, 138.1, 137.8, 136.7, 129.8, 128.2, 127.7, 127.5, 126.7, 126.2, 125.5, 123.4, 115.1, 34.8, 31.5; ATR (cm^−1^): 1660 ν(C=O); HRMS (FD) calcd for C_45_H_41_NOS_2_ *m/z*: 675.2630, found: 675.2614; elemental analysis calcd (%) for C_45_H_41_NOS_2_ + 0.05 CH_2_Cl_2_: C 79.55, H 6.09, N 2.06, found: C 79.18, H 6.17, N 1.90.

*2-((5-(4-(Diphenylamino)phenyl)thieno[3,2-b]thiophen-2-yl)methylene)malononitrile* (**Ib**): To a round flask containing **Ia** (46.3 mg, 0.112 mmol), 4.5 mL of toluene were added and the solution was stirred for 5 min. Then malononitrile (14.5 mg, 0.220 mmol), 3.0 mL of absolute EtOH and a catalytic amount of β-alanine were added and the mixture was warmed up to reflux for 24 h. Solvents were evaporated and the crude was dissolved in a small amount of CH_2_Cl_2_ and purified by chromatography column (CH_2_Cl_2_) to yield 46.0 mg of **Ib** as a dark powder (91%). DSC > 158 °C (decomp.); ^1^H-NMR (600 MHz, acetone-*d*_6_): δ 8.46 (s, 1H), 8.22 (s, 1H), 7.88 (s, 1H), 7.68 (d, *J* = 8.7 Hz, 2H), 7.38 (d, *J* = 8.4 Hz, 2H), 7.36 (d, *J* = 8.4 Hz, 2H), 7.13–7.17 (m, 6H), 7.05 (d, *J* = 8.7 Hz, 2H); ^13^C-NMR (150 MHz, CDCl_3_): δ 155.8, 150.9, 149.7, 149.5, 147.0, 138.5, 135.8, 130.5, 129.7, 127.3, 126.3, 125.5, 124.2, 122.3, 114.6, 114.1, 113.9, 75.4; ATR (cm^−1^): 2213 ν(C≡N); HRMS (FD) calcd for C_28_H_17_N_3_S_2_ *m/z*: 459.0864, found: 459.0879; elemental analysis calcd (%) for C_28_H_17_N_3_S_2_: C 73.17, H 3.73, N 9.14, found: C 72.84, H 3.67, N 9.19.

*2-((5-(4-(Bis(4-bromophenyl)amino)phenyl)thieno[3,2-b]thiophen-2-yl)methylene)malononitrile* (**IIb**): To a round flask containing **IIa** (31.8 mg, 0.056 mmol) in 42.0 mL of absolute EtOH, malononitrile (8.4 mg, 0.123 mmol) and a catalytic amount of β-alanine were added and the mixture was warmed up to reflux for 48 h. After cooling down to room temperature, filtration and washing with pentane, 30.1 mg of **IIb** as a dark powder (87%) were obtained. DSC > 183 °C (decomp.); ^1^H-NMR (300 MHz, DMSO-*d*_6_): δ 8.73 (s, 1H), 8.25 (s, 1H), 7.98 (s, 1H), 7.70 (d, *J* = 8.7 Hz, 2H), 7.52 (d, *J* = 8.8 Hz, 4H), 7.07 (d, *J* = 8.7 Hz, 2H), 7.04 (d, *J* = 8.8 Hz, 4H); ^13^C-NMR (150 MHz, CD_2_Cl_2_): δ 155.3, 151.5, 149.7, 148.7, 146.2, 139.0, 136.4, 133.0, 131.3, 128.0, 127.8, 126.9, 123.6, 117.0, 115.0, 114.9, 114.2, 76.0; ATR (cm^−1^): 2218 ν(C≡N); HRMS (FD) calcd for C_28_H_15_Br_2_N_3_S_2_ *m/z*: 614.9074, found: 614.9052; elemental analysis calcd (%) for C_28_H_15_Br_2_N_3_S_2_ + 0.05 C_5_H_12_: C 54.64, H 2.53, N 6.77, found: C 55.02, H 2.26, N 6.69.

*2-((5-(4-(Bis(4-bromophenyl)amino)phenyl)-6-bromothieno[3,2-b]thiophen-2yl)methylene)malononitrile* (**II’b**): To a round flask containing **II’a** (40.0 mg, 0.062 mmol) in 85.0 mL of absolute EtOH, malononitrile (4.5 mg, 0.068 mmol) and a catalytic amount of β-alanine were added and the mixture was warmed up to reflux for 72 h. EtOH was distilled until appearing of solid, then the crude was slowly cooled to room temperature and left for 3 h. Filtration and washing with pentane gave 28.2 mg of **II’b** as a dark powder (66%). DSC > 230 °C (decomp.); ^1^H-NMR (400 MHz, CD_2_Cl_2_): δ 7.98 (s, 1H), 7.90 (s, 1H), 7.66 (d, *J* = 8.8 Hz, 2H), 7.44 (d, *J* = 8.8 Hz, 4H), 7.11 (d, *J* = 8.8 Hz, 2H), 7.03 (d, *J* = 8.8 Hz, 4H); ^13^C-NMR (150 MHz, CD_2_Cl_2_): δ 151.5, 150.8, 148.8, 148.1, 146.1, 136.3, 136.1, 133.1, 131.4, 130.3, 127.1, 126.4, 122.8, 117.2, 114.5, 113.7, 99.7, 77.8; ATR (cm^−1^): 2225 ν(C≡N); HRMS (FD) calcd for C_28_H_14_Br_3_N_3_S_2_ *m/z*: 692.8180, found: 692.8179; elemental analysis calcd (%) for C_28_H_14_Br_3_N_3_S_2_: C 48.30, H 2.03, N 6.04, found: C 47.99, H 1.86, N 6.05.

*2-((5-(4-(Bis(4'-(tert-butyl)-[1,1'-biphenyl]-4-yl)amino)phenyl)thieno[3,2-b]thiophen-2yl)methylene)malononitrile* (**IIIb**): To a round flask containing **IIIa** (30.0 mg, 0.044 mmol) in 7.0 mL of toluene, malononitrile (27.0 mg, 0.409 mmol), 4.0 mL of absolute EtOH and β-alanine were added. The mixture was warmed up to reflux for 24 h. Solvents were evaporated and the crude was dissolved in CH_2_Cl_2_ and purified by chromatography column (CH_2_Cl_2_) to yield 24.0 mg of **IIIb** as a dark powder (75%). DSC > 145 °C (decomp.); ^1^H-NMR (300 MHz, acetone-*d*_6_): δ 8.46 (s,1H), 8.22 (s,1H), 7.89 (s,1H), 7.72 (d, *J* = 8.6 Hz, 2H), 7.66 (d, *J* = 8.5 Hz, 4H), 7.59 (d, *J* = 8.3 Hz, 4H), 7.49 (d, *J* = 8.3 Hz, 4H), 7.23 (d, *J* = 8.5 Hz, 4H), 7.15 (d, *J* = 8.6 Hz, 2H), 1.34 (s, 18H); ^13^C-NMR (75 MHz, acetone-*d*_6_): δ 155.7, 153.2, 150.9, 150.2, 149.8, 146.8, 139.1, 138.1, 137.4, 137.2, 133.6, 128.7, 128.2, 127.9, 127.1, 126.7, 126.3, 123.5, 116.0, 115.4, 114.8, 75.4, 35.1, 31.6; ATR (cm^−1^): 2216 ν(C≡N); HRMS (FD) calcd for C_48_H_41_N_3_S_2_ *m/z*: 723.2742, found: 723.2708; elemental analysis calcd (%) for C_48_H_41_N_3_S_2_ + 0.04 CH_2_Cl_2_: C 79.32, H 5.69, N 5.78, found: C 79.63, H 5.71, N 5.75.

*5-((5-(4-(Diphenylamino)phenyl)thieno[3,2-b]thiophen-2-yl)methylene)-1,3-diethyl-2-thioxodihydropyrimidine-4,6(1H,5H)-dione* (**Ic**): To a round flask containing **Ia** (30.0 mg, 0.073 mmol) in 25.0 mL of absolute EtOH, 1,3-diethyl-2-thiobarbituric acid (16.1 mg, 0.080 mmol) were added and the mixture was warmed up to reflux for 24 h. Filtration of the precipitate obtained after cooling to room temperature, gave 36.0 mg of **Ic** as a dark powder (83%). DSC > 180 °C (decomp.); ^1^H-NMR (600 MHz, CD_2_Cl_2_): δ 8.71 (s, 1H), 8.09 (s, 1H), 7.57 (d, *J* = 8.7 Hz, 2H), 7.50 (s, 1H), 7.30–7.34 (m, 4H), 7.10–7.17 (m, 6H), 7.06 (d, *J* = 7.0 Hz, 2H), 4.59 (q, *J* = 7.0 Hz, 2H), 4.55 (q, *J* = 7.0 Hz, 2H), 1.32 (t, *J* = 7.0 Hz, 3H), 1.29 (t, *J* = 7.0 Hz, 3H); ^13^C-NMR (150 MHz, CD_2_Cl_2_): δ 179.2, 161.3, 160.2, 156.6, 156.4, 150.0, 149.8, 147.3, 139.4, 139.0, 138.1, 129.9, 127.7, 126.9, 125.8, 124.5, 122.4, 114.8, 110.3, 44.2, 43.5, 12.6, 12.5; ATR (cm^−1^): 1655 ν(C=O); HRMS (FD) calcd for C_33_H_27_N_3_O_2_S_3_ *m/z*: 593.1265, found: 593.1268; elemental analysis calcd (%) for C_33_H_27_N_3_O_2_S_3_: C 66.75, H 4.58, N 7.08, found: C 66.53, H 4.62, N 6.73.

*5-((5-(4-(Bis(4-bromophenyl)amino)phenyl)thieno[3,2-b]thiophen-2-yl)methylene)-1,3-diethyl-2-thioxodihydropyrimidine-4,6(1H,5H)-dione* (**IIc**): To a round flask containing **IIa** (35.0 mg, 0.061 mmol) in 40.0 mL of absolute EtOH, 1,3-diethyl-2-thiobarbituric acid (12.9 mg, 0.064 mmol) and a catalytic amount of β-alanine were added and the mixture was warmed up to reflux for 24 h. Filtration of the precipitate obtained after cooling to room temperature, gave 39.5 mg of **IIc** as a dark powder (86%). DSC > 250 °C (decomp.); ^1^H-NMR (600 MHz, CD_2_Cl_2_): δ 8.72 (s, 1H), 8.10 (s, 1H), 7.61 (d, *J* = 8.7 Hz, 2H), 7.53 (s, 1H), 7.43 (d, *J* = 8.8 Hz, 4H), 7.08 (d, *J* = 7.0 Hz, 2H), 7.02 (d, *J* = 8.8 Hz, 4H), 4.59 (q, *J* = 7.0 Hz, 2H), 4.56 (q, *J* = 7.0 Hz, 2H ), 1.32 (t, *J* = 7.0 Hz, 3H), 1.29 (t, *J* = 7.0 Hz, 3H); ^13^C-NMR (150 MHz, CD_2_Cl_2_): δ 179.2, 161.3, 160.2, 156.1, 155.9, 150.1, 148.7, 146.2, 139.6, 139.1, 138.0, 133.0, 128.2, 127.9, 126.9, 123.5, 117.0, 115.4, 110.6, 44.2, 43.5, 12.6, 12.5; ATR (cm^−1^): 1660 ν(C=O); HRMS (FD) calcd for C_33_H_25_Br_2_N_3_O_2_S_3_ *m/z*: 748.9476, found: 748.9475; elemental analysis calcd (%) for C_33_H_25_Br_2_N_3_O_2_S_3_: C 52.74, H 3.35, N 5.59, found: C 53.06, H 3.11, N 5.54.

*5-((5-(4-(Bis(4-bromophenyl)amino)phenyl)-6-bromothieno[3,2-b]thiophen-2-yl)methylene)-1,3-diethyl-2-thioxodihydropyrimidine-4,6(1H,5H)-dione* (**II’c**): To a round flask containing **II’a** (30.0 mg, 0.046 mmol) in 65.0 mL of absolute EtOH, 1,3-diethyl-2-thiobarbituric acid (10.2 mg, 0.051 mmol) and a catalytic amount of β-alanine were added and the mixture was warmed up to reflux for 24 h. After cooling to room temperature the solid was filtered, washed with pentane and dried, leading to 31.0 mg of **II’c** as a dark solid (81%). DSC > 265 °C (decomp.); ^1^H-NMR (300 MHz, CD_2_Cl_2_): δ 8.73 (s,1H), 8.14 (s, 1H), 7.69 (d, *J* = 8.9 Hz, 2H), 7.44 (d, *J* = 8.9 Hz, 4H), 7.11 (d, *J* = 8.8 Hz, 2H), 7.04 (d, *J* = 8.8 Hz, 4H), 4.60 (q, *J* = 7.0 Hz, 2H), 4.56 (q, *J* = 7.0 Hz, 2H), 1.34 (t, *J* = 7.0 Hz, 3H), 1.29 (t, *J* = 7.0 Hz, 3H); ^13^C-NMR (150 MHz, CD_2_Cl_2_): δ 179.2, 161.3, 160.2, 156.1, 155.9, 150.1, 148.7, 146.2, 139.6, 139.1, 138.0, 133.0, 128.2, 127.9, 126.9, 123.5, 117.0, 115.4, 110.6, 44.2, 43.5, 12.6, 12.5; ATR (cm^−1^): 1687 ν(C=O); HRMS (FD) calcd for C_33_H_24_Br_3_N_3_O_2_S_3_ *m/z*: 826.8581, found: 826.8584; elemental analysis calcd (%) for C_33_H_24_Br_3_N_3_O_2_S_3_: C 47.73, H 2.91, N 5.06, found: C 47.68, H 2.80, N 5.05.

*5-((5-(4-(Bis(4'-(tert-butyl)-[1,1'-biphenyl]-4-yl)amino)phenyl)thieno[3,2-b]thiophen-2-yl)methylene)-1,3-diethyl-2-thioxodihydropyrimidine-4,6(1H,5H)-dione* (**IIIc**): To a round flask containing **IIIa** (40.0 mg, 0.059 mmol) in 5.0 mL of toluene, 6.6 mL of absolute EtOH, 1,3-diethyl-2-thiobarbituric acid (13.0 mg, 0.065 mmol) and a catalytic amount of β-alanine were added and the mixture was warmed up to reflux for 24 h. Solvents were evaporated until 0.5 mL then, 10 mL of absolute EtOH were added obtaining a dark green precipitate which was collected by means of filtration. After washing with pentane and drying, 39.0 mg of **IIIc** (77%) as a dark green solid were obtained. DSC > 230 °C (decomp.); ^1^H-NMR (600 MHz, CD_2_Cl_2_): δ 8.72(s, 1H), 8.10 (s, 1H), 7.62 (d, *J* = 8.8 Hz, 2H), 7.58 (d, *J* = 8.6 Hz, 4H), 7.55 (d, *J* = 8.5 Hz, 4H), 7.53 (s, 1H), 7.47 (d, *J* = 8.5 Hz, 4H), 7.24 (d, *J* = 8.6 Hz, 4H), 7.17 (d, *J* = 8.8 Hz, 2H), 4.59 (q, *J* = 7.0 Hz, 2H), 4.56 (q, *J* = 7.0 Hz, 2H), 1.36 (s, 18H), 1.32 (t, *J* = 7.0 Hz, 3H), 1.29 (t, *J* = 7.0 Hz, 3H); ^13^C-NMR (150 MHz, CD_2_Cl_2_): δ 179.2, 161.3, 160.2, 156.5, 156.4, 150.7, 150.0, 149.5, 146.2, 139.4, 139.0, 138.0, 137.8, 137.0, 128.3, 127.8, 127.2, 126.7, 126.2, 125.8, 122.9, 115.0, 110.4, 44.2, 43.5, 34.8, 31.5, 12.6, 12.5; ATR (cm^−1^): 1646 ν(C=O); HRMS (FD) calcd for C_53_H_51_N_3_O_2_S_3_ *m/z*: 857.3143, found: 857.3134; elemental analysis calcd (%) for C_53_H_51_N_3_O_2_S_3_: C 74.18, H 5.99, N 4.90, found: C 73.82, H 5.80, N 4.87.

### 3.3. Photophysical Study

All photophysical studies have been performed with freshly prepared air-equilibrated solutions at room temperature (298 K). UV/Vis absorption spectra were recorded on a Jasco V-670 spectrophotometer (Jasco, Lisses, France). Steady state was carried out on a Fluoromax spectrofluorometer and time resolved fluorescence measurements were carried out on a Fluorolog spectrofluorometer (Horiba Jobin Yvon, Long Jumeau, France). Fully corrected emission spectra were obtained under excitation at the wavelength of the absorption maximum. Fluorescence quantum yields of dilute dye solutions and of the FONs suspensions were measured according to literature procedures using Nile Blue (Φ_f_ = 0.27 at 541 nm in EtOH), Cresyl Violet (Φ_f_ = 0.54 at 570 nm in MeOH), Indocyanine Green (Φ_f_ = 0.11 at 678 nm in DMSO), Rhodamine-6G (Φ_f_ = 0.94 at 488 nm in EtOH) or Fluorescein (Φ_f_ = 0.9 at 474 nm in NaOH 0.1 M) depending on the emission range [40,41]. The reported fluorescence quantum yield values obtained via this method are within ± 0.02. Fluorescence decays were measured in a time-correlated single photon counting (TCSPC) configuration, under excitation from a NanoLED (370 nm or 455 nm). The instrument response was determined by measuring the light scattered by a Ludox suspension. The lifetime values were obtained from the reconvolution fit analysis of the decays profiles; the quality of the fits was judged by the reduced χ^2^ value (0.9 < χ^2^ < 1.1). The reported lifetimes are within ± 0.1 ns.

### 3.4. Two-Photon Absorption

Two-photon absorption cross sections (σ_2_) were determined from the two-photon excited fluorescence (TPEF) (σ_2_Φ_f_) and the fluorescence quantum yield (Φ_f_). TPEF cross sections were measured relative to Fluorescein in 0.01 M aqueous NaOH in the 690–1000 nm spectral range and relative to Nile Red in DMSO in the 1000–1160 nm spectral range, using the method described by Xu and Webb [23] and the appropriate solvent-related refractive index corrections. Reference values between 700 and 715 nm for Fluorescein were taken from literature. The quadratic dependence of the fluorescence intensity on the excitation power was checked at all wavelengths. Measurements were conducted using an excitation source delivering fs pulses. This allows avoiding excited-state absorption during the pulse duration a phenomenon that has been shown to lead to overestimated two-photon absorption cross-section values. To scan the 680–1080 nm range, a Nd:YVO4-pumped Ti:Sapphire oscillator was used generating 140 fs pulses at a 80 MHz rate. To scan the 1000–1400 nm range, an OPO (PP-BBO) was added to the setup to collect and modulate the output signal of the Ti:Sapphire oscillator. The excitation was focused into the cuvette through a microscope objective (10X, NA 0.25). The fluorescence was detected in epifluorescence mode via a dichroic mirror (Chroma 675dcxru) and a barrier filter (Chroma e650sp-2p) by a compact CCD spectrometer module BWTek BTC112E. Total fluorescence intensities were obtained by integrating the corrected emission. The experimental uncertainty of the absorption cross-section values determined from this method has been estimated to be ±10%.

### 3.5. Structural and Morphological Characterization

TEM was carried out using a HITACHI H7650 (Schaumburg, IL, USA). The copper grid coated with a carbon membrane was pretreated using the Glow discharge technique to yield positively charged hydrophilic carbon surface to allow stronger interaction between the sample and the grid itself and thus easier imaging. One droplet of the dilute aqueous suspension was deposited on the grid and the excess liquid was drowning off with a paper. A staining procedure using uranyl acetate was then used to enhance the contrast.

Zeta-potential analysis was performed using a SZ-100Z Horiba instrument (Horiba Jobin Yvon, Long Jumeau, France), 20 measurements were realized for each sample according to a predefined operating procedure.

The global concentration of the dye in the FONs’ suspension was determined by taking an aliquot of the suspension, which was further lyophilized then dissolved in the same volume of CHCl_3_. The concentration was then derived from the absorbance and the value of the molar extinction coefficient in CHCl_3_ by using the Beer-Lambert law.

## Figures and Tables

**Table 1:** Experimental conditions for Knoevenagel condensations.

Compd.	R_1_, R_2_	Eq ^1^	Solvents	Duration (h)	Yield (%)
**Ib**	H, H	2.0	Tol:EtOH = 1.5:1.0	24	91
**IIb**	Br, H	2.2	EtOH	48	87
**II’b**	Br, Br	1.1	EtOH	72	66
**IIIb**	*tert*-butyl-C_6_H_4_, H	9.3	Tol:EtOH = 1.7:1.0	24	75
**Ic**	H, H	1.1	EtOH	24	83
**IIc**	Br, H	1.0	EtOH	24	86
**II’c**	Br, Br	1.1	EtOH	24	81
**IIIc**	*tert*-butyl-C_6_H_4_, H	1.1	Tol:EtOH = 1.0:1.3	24	77

^1^ Number of equivalent used for malononitrile (**b**) or 1,3-diethyl-2-thiobarbituric acid (**c**).

**Table 2:** One-photon and two-photon photophysical data of derivatives **I**–**IIIa**–**c** in CHCl_3_ solution.

Compd.	λ_max_^1PA^ (nm)	ε^max^ (10^4^ M^−1^·cm^−1^)	λ_max_^em^ (nm)	Φ_f_	Stokes Shift (10^3^ cm^−1^)	ε^max^Φ_f_ (10^4^ M^−1^·cm^−1^)	τ (ns)	k_r_ (10^9^ s^−1^)	k_nr_ (10^9^ s^−1^)	λ_max_^2PA^ (nm)	σ_2_^max^ (GM)
**Ia**	417	3.9	534	0.84	5.2	3.3	2.8	0.30	0.06	830	240
**IIa**	409	3.7	526	0.84	5.4	3.1	2.5	0.34	0.06	820	330
**II’a**	401	2.9	538	0.76	6.3	2.2	2.6	0.29	0.09	820	314
**IIIa**	429	3.9	582	0.73	6.1	2.8	3.3	0.22	0.08	890	480
**Ib**	511	4.2	652	0.47	4.2	2.0	2.6	0.18	0.20	1050	1164
**IIb**	495	5.4	629	0.31	4.3	1.7	1.5	0.21	0.46	960	934
**II’b**	482	4.5	638	0.42	5.1	1.9	2.4	0.18	0.24	960	862
**IIIb**	525	4.5	700	0.24	4.8	1.1	1.9	0.13	0.40	1060	1402
**Ic**	563	6.0	712	0.37	3.7	2.2	1.9	0.19	0.33	1110	1194
**IIc**	547	6.8	681	0.28	3.6	1.9	1.2	0.23	0.60	1110	1072
**II’c**	522	5.5	694	0.30	4.7	1.65	1.6	0.19	0.44	1060	802
**IIIc**	576	5.5	755	0.06	4.1	0.33	0.6	0.10	1.57	1120	2002

**Table 3:** Structural characterization of FONs made from push-pull derivatives **I**–**IIIa**–**c**.

Compd.	d_TEM_ (nm)	ζ-Potential (mV)	N ^1^ (10^4^)
**Ia**	42	−70	5.7
**IIa**	37	−85	2.8
**II’a**	35	−74	2.0
**IIIa**	27	−72	0.9
**Ib**	21	−74	0.6
**IIb**	39	−75	3.0
**II’b**	43	−56	3.5
**IIIb**	32	−79	1.5
**Ic**	30	−67	1.4
**IIc**	29	−75	1.0
**II’c**	26	−70	0.6
**IIIc**	32	−72	1.2

^1^ Estimated number of dyes subunits *per* FON based on their size.

**Table 4:** One-photon and two-photon photophysical data of dyes **I**–**IIIa**–**c** as FONs chromophoric subunits in deionized water.

Compd.	λ_abs_^max^(Δλ^max^) ^1^ (nm)	ε^max^(Δε^max^) ^2^ (10^4^M^−1^·cm^−1^)	λ_max_^em^(Δλ^max^) ^3^ (nm)	Φ_f_	Stokes Shift (10^3^∙cm^−1^)	τ_1_/τ_2_ (ns)	λ_max_^2PA^ (nm)	σ_2_^max^(Δσ_2_^max^) ^4^ (GM)
**Ia**	410 (−7)	3.4 (−0.5)	557 (+23)	0.05	6.4	1.2 (0.46)/3.9 (0.54)	910	395 (+155)
**IIa**	407 (−2)	3.6 (−0.1)	541 (+15)	0.05	6.1	0.9 (0.57)/2.8 (0.43)	820 890	457 (+127) 557 (+227)
**II’a**	401 (0)	2.5 (−0.4)	540 (+2)	0.04	6.4	0.8 (0.71)/2.4 (0.29)	830	165 (−149)
**IIIa**	424 (−5)	3.3 (−0.6)	570 (−12)	0.06	6.0	0.9 (0.59)/3.8 (0.41)	910	182 (−298)
**Ib**	476 (−35)	3.5 (−0.7)	719 (+67)	0.01	7.1	1.5 (0.58)/4.0 (0.42)	1070	300 (−864)
**IIb**	478 (−17)	6.1 (+0.7)	711 (+82)	0.01	6.9	1.1 (0.73)/3.5 (0.27)	1060	921 (−13)
**II’b**	478 (−4)	/	702 (+64)	0.01	6.7	1.1 (0.84)/3.5 (0.16)	1060	739 (−123)
**IIIb**	507 (−18)	5.0 (+0.5)	714 (+14)	0.02	5.7	1.5 (0.50)/4.6 (0.50)	1080	726 (−676)
**Ic**	517 (−46)	5.2 (−0.8)	754 (+42)	0.004	6.1	0.9 (0.80)/2.7 (0.20)	/	/
**IIc**	509 (−38)	5.5 (−1.3)	752 (+71)	0.007	6.3	1.0 (0.80)/2.9 (0.20)	/	/
**II’c**	502 (−20)	5.2 (−0.3)	725 (+30)	0.006	6.1	0.7 (0.84)/2.2 (0.16)	/	/
**IIIc**	541 (−35)	4.9 (−0.6)	749 (−6)	0.005	5.1	1.0 (0.76)/3.0 (0.24)	/	/

^1^ Shift in λ_abs_^max^ upon molecular confinement within FONs. ^2^ Variation in molar extinction coefficients upon molecular confinement within FONs. ^3^ Shift in λ_em_^max^ upon molecular confinement within FONs. ^4^ Variation in two-photon absorption cross-sections upon molecular confinement within FONs.

**Table 5:** Luminescence properties including one-photon and two-photon brightness of FONs.

Compd.	λ_max_^1PA^ (nm)	λ_max_^em^ (nm)	FWHM^em^ (10^3^∙cm^−1^)	Φ_f_	ε_max_^NPs^Φ_f_ * (10^8^ M^−1^∙cm^−1^)	λ_max_^2PA^ (nm)	σ_2max_^NPs^Φ_f_ * (10^6^ GM)
**Ia**	410	557	3.1	0.05	1.0	910	1.12
**IIa**	407	541	3.0	0.05	0.5	890	0.76
**II’a**	401	540	3.0	0.04	0.1	830	0.64
**IIIa**	424	570	3.6	0.06	0.4	910	0.22
**Ib**	476	719	2.5	0.01	0.02	1070	0.02
**IIb**	478	711	5.6	0.01	0.2	1060	0.3
**II’b**	478	702	2.6	0.01	/	1060	0.1
**IIIb**	507	714	2.5	0.02	0.4	1080	0.5
**Ic**	517	754	2.5	0.004	0.03	/	/
**IIc**	509	752	2.6	0.007	0.04	/	/
**II’c**	502	725	2.7	0.006	0.04	/	/
**IIIc**	541	749	2.5	0.005	0.02	/	/

* The one-photon (ε_max_^NPs^Φ_f_) and two-photon brightness σ_2max_^NPs^Φ_f_ are those of the nanoparticle (calculated using the average number of dyes subunits per nanoparticle and the ε_max_ and σ_2max_ values of the dye subunits).

**Table 6:** Photophysical properties of core-shell FONs **IIIc@IIa** in water (compared to pure FONs **IIIc** and **IIa**).

FONs	λ_max_^1PA^ (nm)	λ_max_^em^ (nm)	Φ_f_	τ_1_/τ_2_ (ns)
**IIa**	407	541	0.05 ^a^	0.9 (0.57)/2.8 (0.43)
**IIIc**	541	749	0.005 ^b^	1.0 (0.76)/3.0 (0.24)
**IIIc@IIa**	339 417 542	710	0.10 (exc@417 nm) ^b^	0.5 (0.80)/2.1 (0.20) ^c^ 2.2 (0.62)/4.6 (0.38) ^d^

^a^ Fluorescence quantum yield determined using fluorescein in 0.01 M NaOH (Ф_f_ = 0.9) as reference. ^b^ Fluorescence quantum yield determined using Nile Blue in ethanol (Ф_f_ = 0.27) as reference. ^c^ Lifetime of fluorescence emission detected at 530 nm (excitation at 455 nm). ^d^ Lifetime of fluorescence emission detected at 700 nm (excitation at 455 nm).

## Supplementary Materials

Supplementary materials can be accessed at: http://www.mdpi.com/1420-3049/21/9/1227/s1.

## Abbreviations

The following abbreviations are used in this manuscript:

CHO: formyl
DCV: dicyanovinyl
DETB: 1,3-diethyl-2-thiobarbiturate
ICT: internal charge transfer
NIR: near infrared
1PA: one-photon absorption
2PA: two-photon absorption
TPEF: two-photon excited fluorescence
FONs: fluorescent organic nanoparticles
TEM: transmission electron microscopy
