# Supplementary material for: Molecular-Based Fluorescent Nanoparticles Built from Dedicated Dipolar Thienothiophene Dyes as Ultra-Bright Green to NIR Nanoemitters
Source: Molecules. 2016 Sep 14;21(9):1227. doi: 10.3390/molecules21091227 (PMC6273080; doi:10.3390/molecules21091227)
Supplement: Supplementary file 1 [file molecules-21-01227-s001.pdf]

Supplementary Materials: Molecular-Based Fluorescent Nanoparticles Built from Dedicated Dipolar Thienothiophene Dyes as Ultra-Bright Green to NIR Nanoemitters

Cristiano Mastrodonato, Paolo Pagano, Jonathan Daniel, Michel Vaultier and Mireille Blanchard-Desce

Table of Contents

I. Photophysical study of dyes **I–III,a–c** in organic solvents

I.1. Solvatochromism

I.2. Comparison of the one-photon absorption and two-photon absorption properties of dyes **I–IIIa–c** in $CHCl_3$

II. Morphology and stability of FONs

III.1. Morphological characterization of FONs made from dyes **I–IIIa–c** by TEM

III.2. Investigation of FONs stability over time

III. Photophysical properties of FONs: effect of molecular confinement on one-photon absorption

IV. Fluorescence decay of chromophores in $CHCl_3$ solution and FONs in water

I.1. Solvatochromism

Table S1. Solvatochromism of dyes **I–IIIa–c** in organic solvents: maximum absorption and emission wavelengths of dyes dissolved in solvents of increasing polarity.

Cpd	process	Cyclohex	Tol	$CHCl_3$	THF	DCM	Acetone	DMSO
**Ia**	abs	403	410	417	407	415	405	406
	ems	455	477	524	534	573	573	609
**IIa**	abs	396	402	409	399	407	398	398
	ems	435	467	509	526	540	551	589
**II′a**	abs	384	393	391	400	397	388	388
	ems	444	476	528	523	557	580	608
**IIIa**	abs	413	420	416	427	423	413	413
	ems	459	493	549	579	605	605	653
**Ib**	abs	484	499	488	511	505	483	/
	ems	534	584	657	665	680	723	/
**IIb**	abs	469	481	476	496	488	472	472
	ems	520	565	626	647	660	705	740
**II′b**	abs	463	413	458	482	472	453	453
	ems	524	571	638	666	683	729	747
**IIIb**	abs	509	509	497	525	517	496	/
	ems	550	601	696	709	743	766	/
**Ic**	abs	544	547	538	560	554	535	/
	ems	583	627	711	710	740	766	/
**IIc**	abs	517	531	523	545	539	521	521
	ems	565	608	683	690	709	752	810
**II′c**	abs	495	505	495	521	515	494	/
	ems	656	612	691	714	731	779	/
**IIIc**	abs	557	557	546	575	565	540	/
	ems	596	646	757	757	792	/	/

I.2. Comparison of the one-photon absorption and two-photon absorption properties of dyes **I–IIIa–c** in $CHCl_3$.

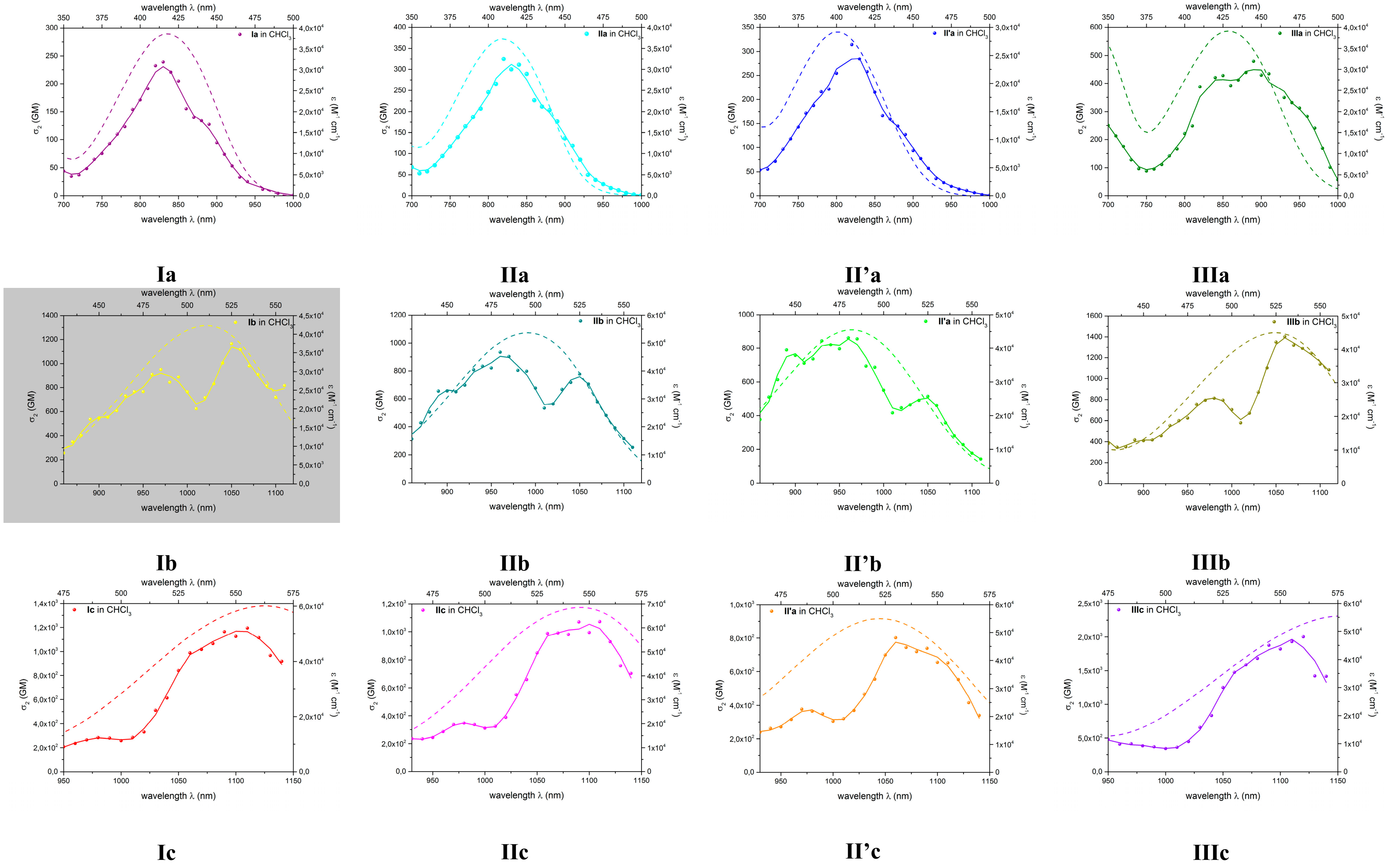

Figure S1. Comparison of the one-photon absorption and two-photon absorption spectra of dyes **I–IIIa–c** in solution in $CHCl_3$**.**

II.1. Morphological characterization of FONs made from dyes **I–IIIa–c** by TEM

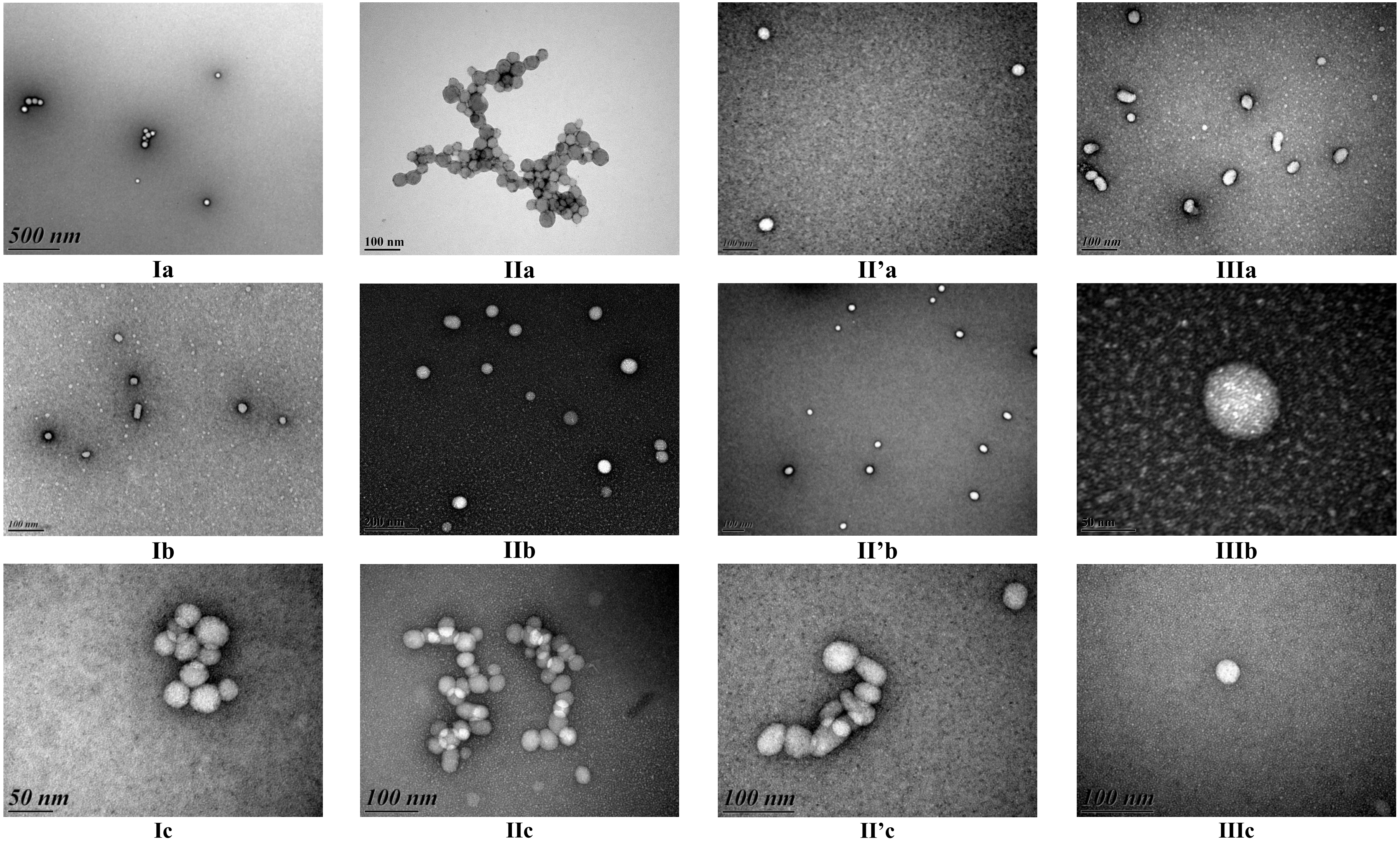

Figure S2. TEM images of FONs made from dyes **I–IIIa–c.**

II.2. Investigation of the FONs stability over time

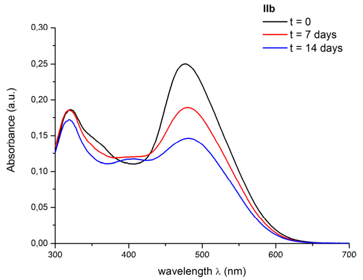

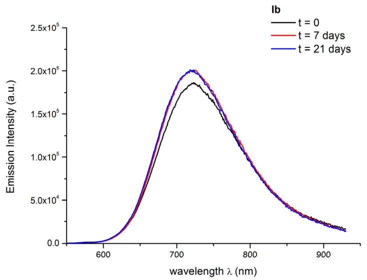

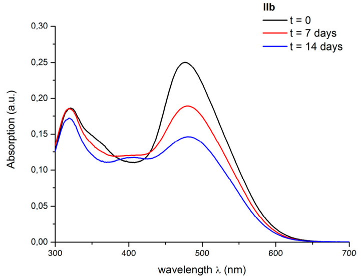

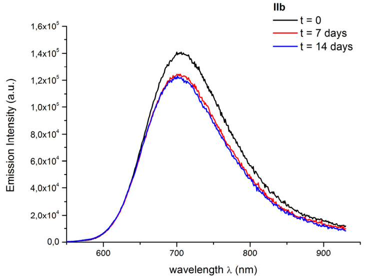

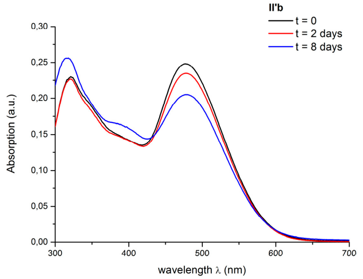

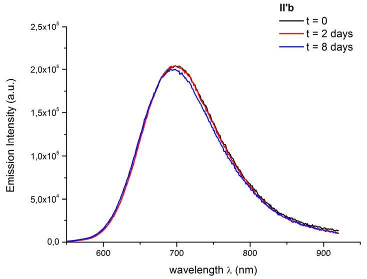

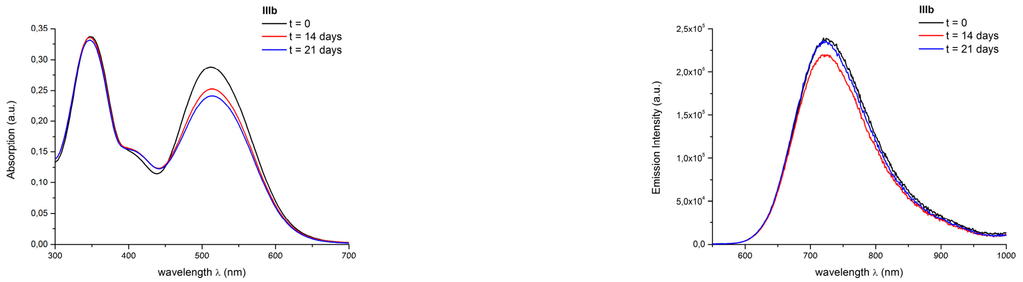

Figure S3. Monitoring of the evolution over time of the absorption and fluorescence spectra of FONs made from dyes **I–IIIb.**

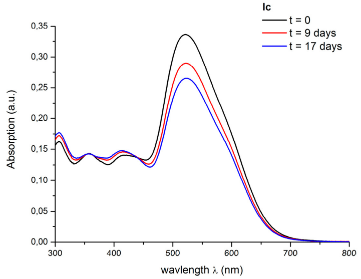
Ic
t = 0
t = 9 days
t = 17 days
Absorption (a.u.)
wavlength λ (nm)

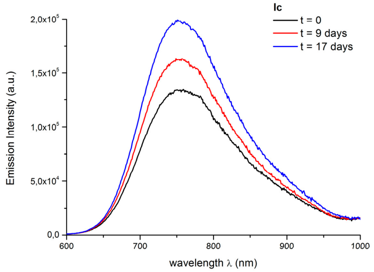
Ic
t = 0
t = 9 days
t = 17 days
Emission Intensity (a.u.)
wavelength λ (nm)

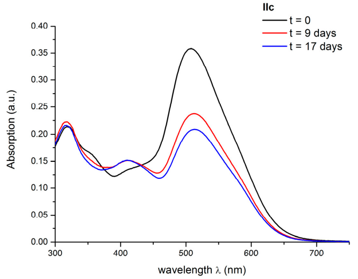
IIc
t = 0
t = 9 days
t = 17 days
Absorption (a.u.)
wavelength λ (nm)

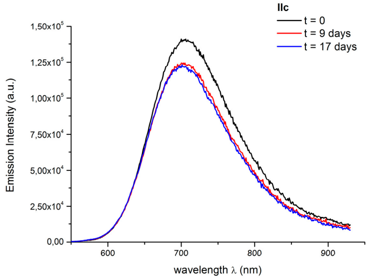
IIc
t = 0
t = 9 days
t = 17 days
Emission Intensity (a.u.)
wavelength λ (nm)

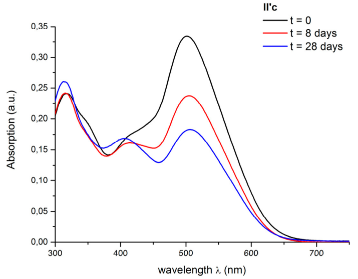
II'c
t = 0
t = 8 days
t = 28 days
Absorption (a.u.)
wavelength λ (nm)

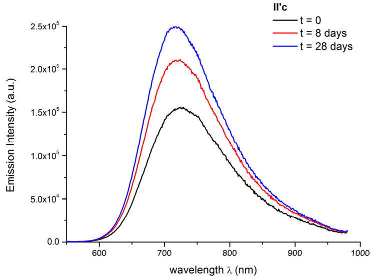
II'c
t = 0
t = 8 days
t = 28 days
Emission Intensity (a.u.)
wavelength λ (nm)

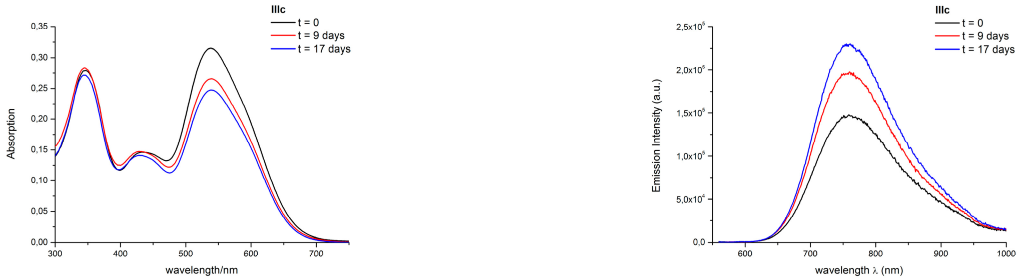

Figure S4. Monitoring of the evolution over time of the absorption and fluorescence spectra of FONs made from dyes **I–IIIc.**

III. Photophysical properties of FONs: effect of molecular confinement on one-photon absorption

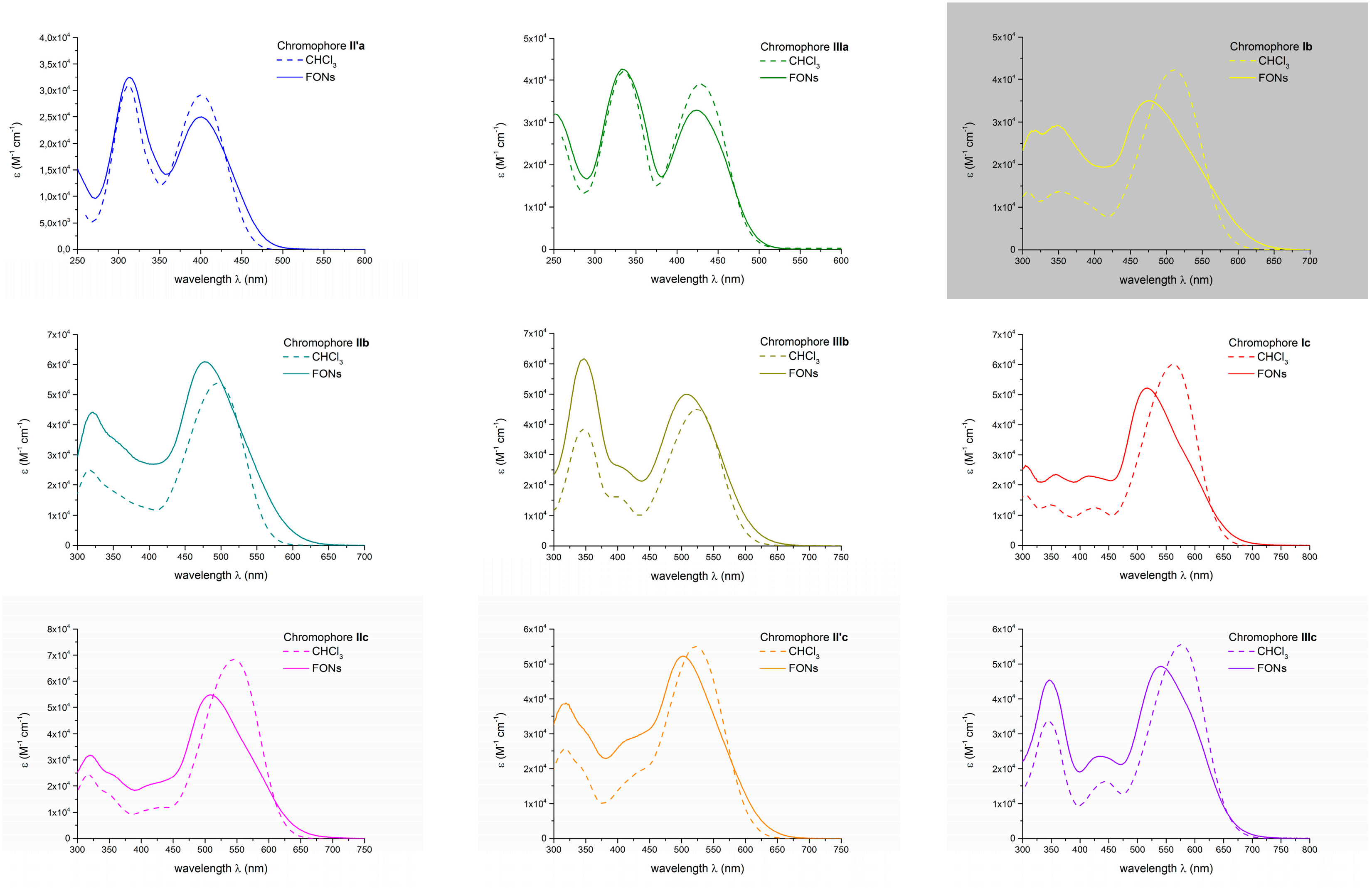

Figure S5. Comparison of the absorption properties of dyes in chloroform and as subunits of FONs.

IV. Fluorescence decay

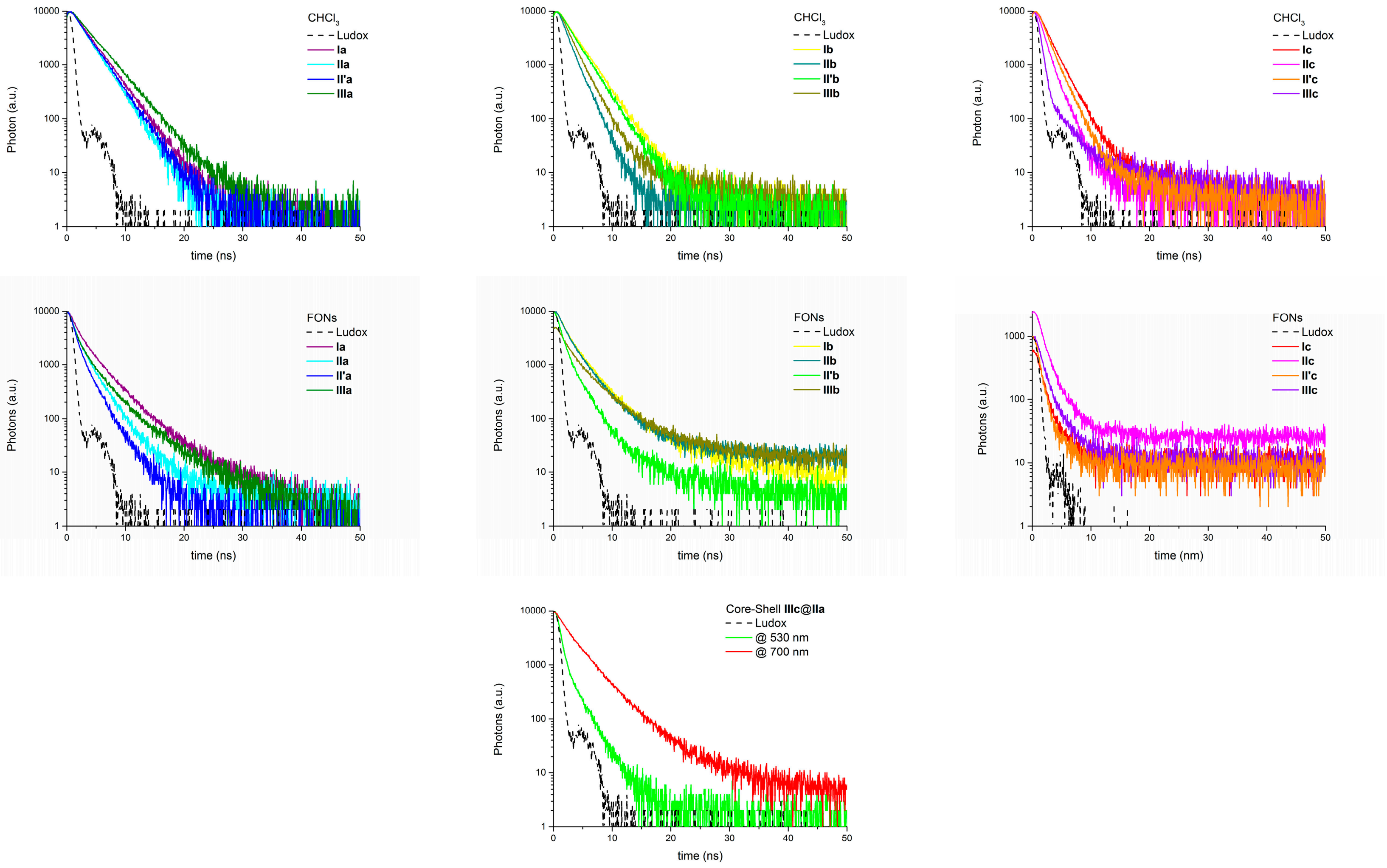

Figure S6. Comparison of the fluorescence decays measured for the three families in $CHCl_3$ solution, in FONs in water and the prepared Core-Shell nanoparticles **IIIc@IIa.**
